# RNase L represses hair follicle regeneration through altered innate immune signaling

**DOI:** 10.1172/JCI172595

**Published:** 2025-02-04

**Authors:** Charles S. Kirby, Nasif Islam, Eric Wier, Martin P. Alphonse, Evan Sweren, Gaofeng Wang, Haiyun Liu, Dongwon Kim, Ang Li, Sam S. Lee, Andrew M. Overmiller, Yingchao Xue, Sashank Reddy, Nathan K. Archer, Lloyd S. Miller, Jianshi Yu, Weiliang Huang, Jace W. Jones, Sooah Kim, Maureen A. Kane, Robert H. Silverman, Luis A. Garza

**Affiliations:** 1 Department of Dermatology and; 2 Cell Biology, Johns Hopkins School of Medicine, Baltimore, Maryland, USA.; 3 Department of Biochemical Engineering, College of Science and Technology, Dongseo University, Busan, South Korea.; 4 Laboratory of Skin Biology, National Institute of Arthritis and Musculoskeletal and Skin Diseases, National Institutes of Health, Bethesda, Maryland, USA.; 5 Department of Plastic Surgery, Johns Hopkins School of Medicine, Baltimore, Maryland, USA.; 6 Department of Pharmaceutical Sciences, School of Pharmacy, University of Maryland, Baltimore, Maryland, USA.; 7 Department of Environment Science and Biotechnology, College of Medical Science, Jeonju University, Jeonju, South Korea.; 8 Department of Cancer Biology, Lerner Research Institute, Cleveland Clinic, Cleveland, Ohio, USA.; 9 Department of Oncology, Johns Hopkins University, Baltimore, Maryland, USA.

**Keywords:** Dermatology, Inflammation, Innate immunity, Skin

## Abstract

Mammalian injury responses are predominantly characterized by fibrosis and scarring rather than functional regeneration. This limited regenerative capacity in mammals could reflect a loss of proregeneration programs or active suppression by genes functioning akin to tumor suppressors. To uncover programs governing regeneration in mammals, we screened transcripts in human participants following laser rejuvenation treatment and compared them with mice with enhanced wound-induced hair neogenesis (WIHN), a rare example of mammalian organogenesis. We found that *Rnasel^–/–^* mice exhibit an increased regenerative capacity, with elevated WIHN through enhanced IL-36α. Consistent with RNase L’s known role to stimulate caspase-1, we found that pharmacologic inhibition of caspases promoted regeneration in an IL-36–dependent manner in multiple epithelial tissues. We identified a negative feedback loop, where RNase L–activated caspase-1 restrains the proregenerative dsRNA-TLR3 signaling cascade through the cleavage of toll-like adaptor protein TRIF. Through integrated single-cell RNA-seq and spatial transcriptomic profiling, we confirmed OAS & IL-36 genes to be highly expressed at the site of wounding and elevated in *Rnasel^–/–^* mouse wounds. This work suggests that RNase L functions as a regeneration repressor gene, in a functional trade off that tempers immune hyperactivation during viral infection at the cost of inhibiting regeneration.

## Abstract 2

Inhibition of the OAS-RNase L pathway promotes wound healing and epithelial regeneration.

## Graphical abstract

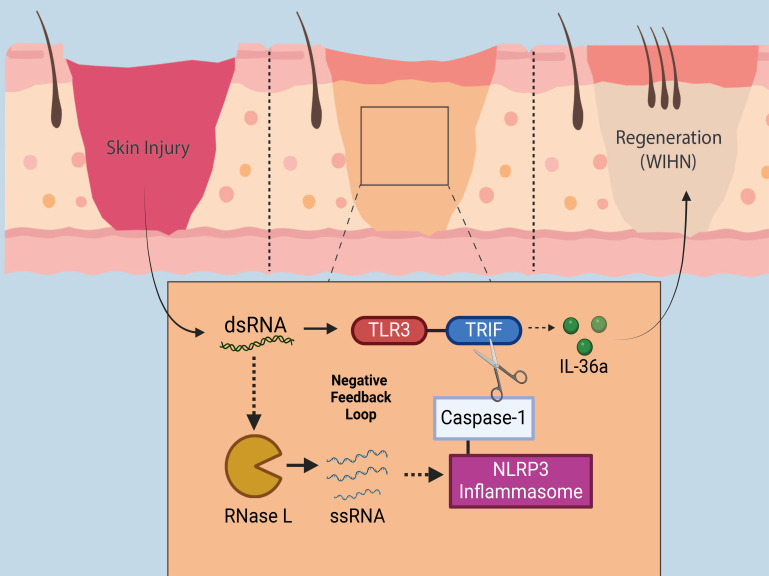

## Introduction

Upon amputation, animals such as urodele amphibians form a dedifferentiated cell cluster, known as the blastema, which coordinates whole appendage regeneration (1, 2). Mammals, by contrast, lack this capacity for epimorphic regeneration. One exception to this is the induction of de novo hair follicles in adult skin after full-thickness excisional wounding in mice and rabbits, a process termed wound-induced hair neogenesis (WIHN) (3, 4). WIHN is characterized by a morphogenic cascade that recapitulates embryogenic events, whereby multipotent keratinocyte progenitors communicate with mesenchymal fibroblasts to create neogenic hair follicles and surrounding skin. Important advances have been made in defining the role of developmental pathways such as Wnt, Shh, and Fgf in driving WIHN (4–6). However, the canonical wound-associated events that activate these developmental pathways have received less attention.

Immune signaling plays a crucial role in the regulation of WIHN (7, 8), but less is known about the upstream mechanisms that receive and modulate these signals. After tissue injury, the sensing of double-stranded RNA (dsRNA) released as a damage-associated molecular pattern (DAMP) has been shown to play an important role in early wound-associated pathways (9–11). Activation of Toll-like Receptor 3 (TLR3) by dsRNA stimulates a regenerative response and triggers the release of proinflammatory signals necessary for WIHN (10). Previous studies have also demonstrated the abundance of other dsRNA sensors during wound regeneration, including the antiviral 2’-5’ oligoadenylate synthetase (OAS) gene family, which is found in mice with high WIHN and in humans after postlaser photo rejuvenation treatment. (10, 12) This highlights a multispecies role for dsRNA sensing in wound repair as well as a gap in knowledge in the activation of dsRNA targets and their effect on regeneration.

Important in antiviral innate immunity, the OAS family are classically known as dsRNA sensors that produce 5’-triphosphorylated 2'-5' adenylyl oligomers that, in turn, activate the endoribonuclease RNase L (13). There is emerging evidence that OASs have functionality independent RNase L activation through the regulation and protection of interferons (14, 15). OASs have also been observed to impact cellular processes beyond antiviral immunity, regulating immune and cell cycle activation during skin cell proliferation and wound repair. (16, 17) However, less is known about how OASs influence cellular processes, particularly during wounding, and whether this is functionally connected to or separate from RNase L. Upon activation, RNase L cleaves both viral and cellular RNA to stimulate antiviral immunity, initiating apoptosis (18), autophagy (19), and the inhibition of cellular migration (20). RNase L plays an important role in host immune defense through the activation of the NLRP3 inflammasome and subsequent activation of the caspase-1 protease (21). Paradoxically, loss of RNase L can lead to the opposite phenomenon, increasing inflammatory cytokine release upon intracellular dsRNA stimulation (22). This suggests that, although RNase L represents a critical control point for innate immunity, the exact nature and direction of this influence can depend on several contextual factors, including cell type. Given the importance of the skin as the first line of defense against viral infection and the centrality of innate immunity, the immunoregulatory function of OAS/RNase L/Caspases remains underexplored within skin biology.

In this manuscript, we sought to test a function of the OAS response by focusing on RNase L as a point of convergence from multiple distinct dsRNA-stimulated OAS family members. We found RNase L to be an important mediator of epithelial damage response signaling via the downstream activation of the NLRP3 inflammasome and caspase cleavage. Surprisingly, rather than stimulating WIHN, as dsRNA does itself, RNase L acted as negative feedback to limit WIHN. Similarly, RNase L–stimulated caspase activity also restrained WIHN, at least partially, through inhibition of the proregenerative cytokine IL-36α. Finally, we define an important contributory mechanism of this negative feedback loop where Caspase-1 protease cleaves TRIF to inhibit TLR signaling and WIHN. This work highlights a nonimmunologic role of dsRNA sensing in regeneration where an intricate control of the outcome is dictated by traditional feed-forward signaling as well as a restraining negative feedback loop.

## Results

### The OAS pathway is upregulated during wound healing in mice and human skin.

**Figure 1:**
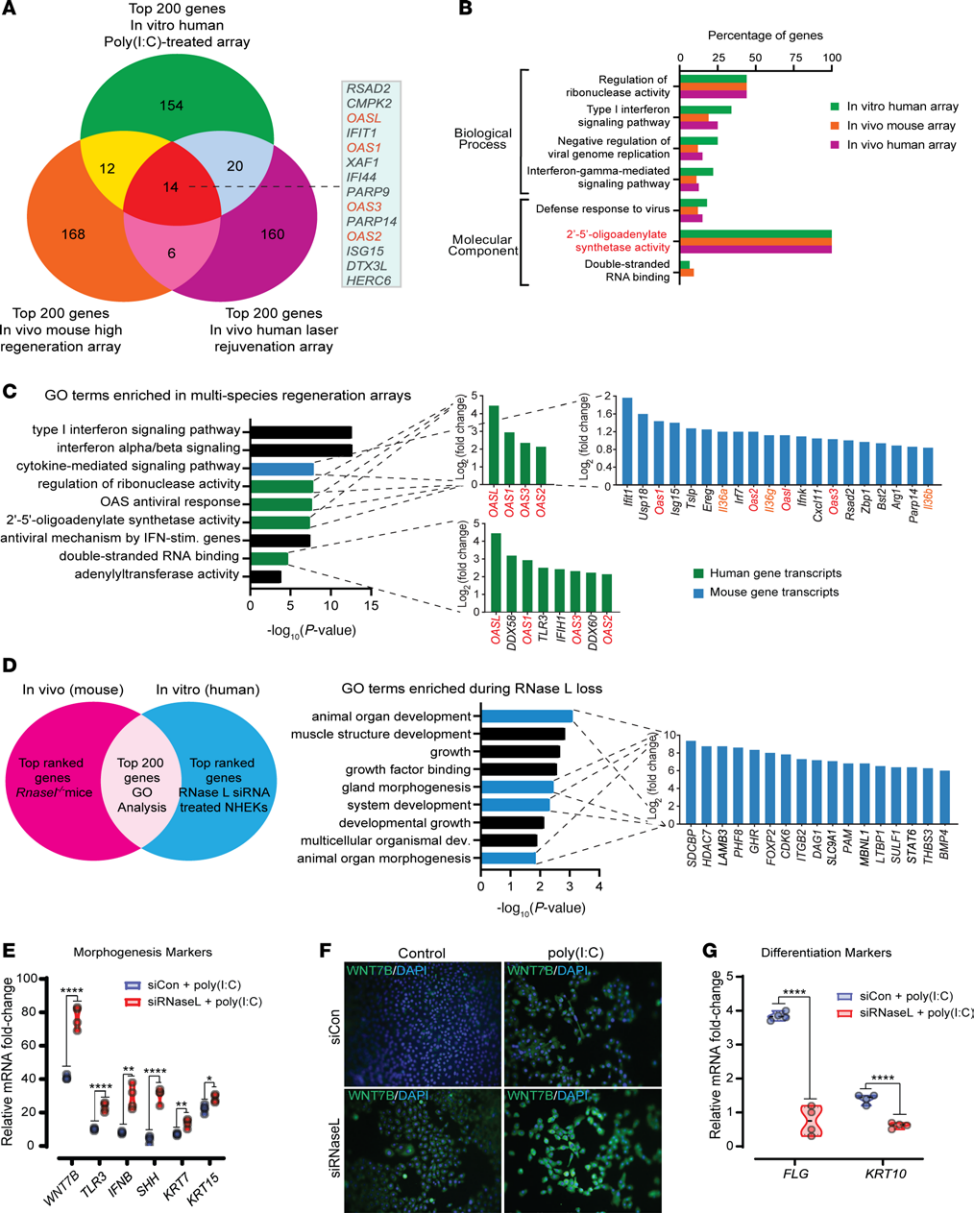
Transspecies dsRNA sensing signature during skin regeneration; RNase L represses regeneration markers. (**A**) 3-way Venn diagram shows a 14-gene overlap present in all of the top 200 genes in microarrays of in vivo WIHN comparing C57BL/6 × FVB × SJL mice (high regeneration strain) versus C57BL/6 mice (low regeneration strain) versus in vivo human clinical trial of participants treated with a rejuvenation laser, with in vitro human keratinocytes treated with dsRNA/Poly (I:C) as a positive control. The in vitro and in vivo human microarrays contain a total of 49,395 annotated transcripts each, and the in vivo murine microarray contains 53,145 transcripts. (**B**) Gene ontology analysis of each of the top 200 gene lists, highlighting the predominance of OAS family members in each data set. (**C**) Gene ontology terms enriched in the 14 overlapping genes from all 3 datasets include the upregulation of OAS family genes. Inset graphs show the gene fold-expression changes from the original microarray for genes present in that category; green and blue indicate mouse and human, respectively. (**D**) Analysis of the ribonuclease RNase L (downstream and activated by OAS), Venn diagram shows the top 200 overlapping genes in *Rnasel^–/–^* mice after wounding (at scab detachment) and human keratinocytes treated with siRNA targeting RNase L. GO categories include multiple developmental pathways. The in vivo microarray data contain 22,206 transcripts and the in vitro RNA-seq data contain 33,264 transcripts. (**E**–**G**) Poly (I:C) treatment (10 μg/mL) of RNase L siRNA transfected human keratinocytes induces multiple morphogenesis markers (**E**; *n* = 4, 2-way ANOVA, *P* < 0.05), including WNT7B (green) shown by immunostaining (original magnification, × 20) (**F**), but inhibits differentiation markers as measured by RT-PCR with fold changes compared with siCon without stimulation (**G**; *n* = 4, 2-way ANOVA, *P* < 0.05).

To define common mechanisms that dictate tissue regeneration, we performed transcriptome analysis for a common injury-mediated rejuvenation signature in humans and mice. We probed the intersection of 3 distinct transcriptome screens and defined the top 200 genes in: (a) WT outbred strains of mice with a high capacity for WIHN (C57BL/6J × FVB × SJL) (10); (b) human participants after laser rejuvenation to ameliorate photoaging (12); and (c) cultured human keratinocytes treated with synthetic dsRNA poly(I):poly(C) (polyI:C) (9) as a positive control. A dramatic overlap signature was observed, including all human members of a class of dsRNA sensors, the 2’-5’-OAS gene family (*OAS1*, *OAS2*, *OAS3*, and *OASL*; Figure 1A and Supplemental Figure 1A; supplemental material available online with this article; https://doi.org/10.1172/JCI172595DS1), which are known to be induced in response to polyI:C in an interferon response (23, 24). Gene ontology analysis confirms that OAS RNA is upregulated in all 3 datasets, both individually (Figure 1B) and together (Figure 1C). These results demonstrate a correlation between OAS expression and regeneration responses in mice and humans.

### RNase L inhibition increases dsRNA stimulation of regeneration markers.

To begin a functional analysis, we tested the effect of RNase L loss-of-function on gene expression in a whole-body deletion mouse model and in vitro after gene silencing in human keratinocytes. Common pathways induced in both contexts of RNase L loss include developmental and morphogenesis pathways (Figure 1D), which are also seen in proteomics analysis (Supplemental Figure 2, A and B). dsRNA is known to enhance regeneration by respectively inducing and inhibiting undifferentiated and differentiated cell states via TLR3 (10). To characterize the polarity of RNase L function in the context of regeneration, we added dsRNA polyI:C to normal adult human keratinocytes with or without RNase L depletion. We found that polyI:C treatment coupled with RNase L loss hyperactivated the expression of morphogenesis and stem cell transcripts *WNT7B, TLR3, SHH* (sonic hedgehog), *KRT15*, and *KRT7* (Figure 1, E and F). This effect was also seen in mouse keratinocytes (Supplemental Figure 3A). In parallel, differentiation-associated transcripts were downregulated, including *FLG* (filaggrin) and *KRT10* (Figure 1G). Previous reports have indicated that inhibition of RNase L can amplify or diminish IFN-β response contingent on the specific cell type (17, 18). We found no discernable differences in IFN mRNA after RNase L depletion itself; however, the simultaneous silencing of RNase L and addition of dsRNA does so in keratinocytes Supplemental Figure 3B) (24, 25).

### RNase L represses WIHN.

**Figure 2:**
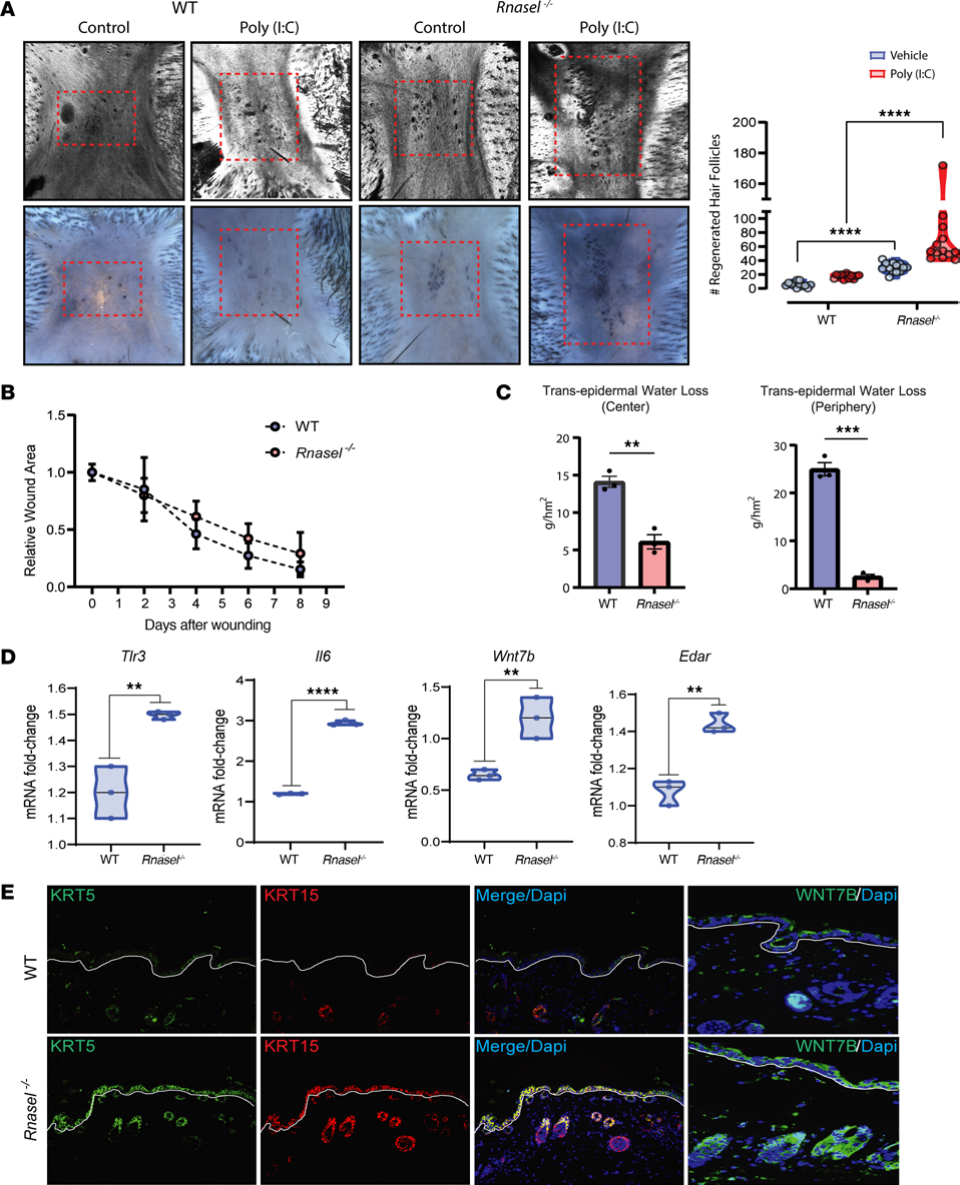
RNase L–loss enhances hair follicle regeneration. (**A**) *Rnasel^–/–^* mice exhibit increased WIHN with an intact superincrease in the presence of poly (I:C) (confocal scanning laser microscopy [CSLM] and Alkaline Phosphatase [AP]staining, images; *n* = 12 WT, 13 WT+PIC and 12 Rnasel^–/–^, 13 Rnasel^–/–^+PIC, 2-way ANOVA, *P* < 0.0001). In each image, the dash red box indicates the area of hair follicle regeneration. (**B**) *Rnasel^–/–^* mice display normal wound closure speed (*n* = 20 WT mice and 17 Rnasel^–/–^ mice). (**C**) Transepidermal water loss (TEWL) was measured in the center and periphery of healed skin at scab detachment day (Would Day 10 [WD10]) for both WT and *Rnasel^–/–^* mice. *Rnasel^–/–^* mice exhibit dramatically lower TEWL measurements, consistent with a postwounding improved barrier compared with WT mice (n=3, 2-tailed unpaired *t* test, *P* < 0.005, *P* = 0.001). (**D**) *Rnasel^–/–^* mice have greater morphogenesis marker gene expression of *Tlr3* (*n* = 3, *P* < 0.01), *Il6* (*n* = 3, *P* < 0.0001), *Wnt7b* (*n* = 3, *P* < 0.01), and *Edar* (*n* = 3, 2-tailed unpaired *t* test, *P* < 0.01) on day of reepithelialization as measured by qRT-PCR. (**E**) Unwounded skin of *Rnasel^–/–^* mice shows increased protein expression of stem cell markers KRT5 (green) and KRT15 (red) and morphogenesis marker WNT7B (green) shown by immunofluorescence (original magnification, × 20).

To investigate the role of RNase L in the regeneration of hair follicles, we wounded *Rnasel^–/–^* and strain-matched control mice and measured WIHN (9, 10, 26). Consistent with the observed changes in gene expression above, *Rnasel^–/–^* mice exhibited enhanced regenerative capability, seen by a greater abundance of de novo hair follicles (Figure 2A). Despite a higher baseline, *Rnasel^–/–^* mice maintained regenerative response capacity after addition of exogenous dsRNA. Given the established role of poly I:C to increase WIHN, this additive effect demonstrates a distinct role of RNase L to suppress regeneration (Figure 2A). Interestingly wound closure speed was not significantly affected by Rnase L loss (Figure 2B and Supplemental Figure 4A), however there is a notable enhancement of barrier restoration in *Rnasel^–/–^* mice (Figure 2C). This may partially be explained by RNase L’s role as an inhibitor of cell migration (20). Additionally unwounded *Rnasel^–/–^* mouse tissue exhibits reduced hypodermis thickness, potentially attributable to RNase L’s observed role in adipogenesis (Supplemental Figure 5A) (27).

During wound reepithelialization, *Rnasel^–/–^* mice express higher levels of morphogenesis transcripts (*Tlr3*, *Il6*, *Wnt7b*, and *Edar*), consistent with our in vitro findings in human and mouse keratinocytes (Figure 1E, Figure 2D, and Supplemental Figure 3A). Skin stem cell markers KRT5 and KRT15 are upregulated in unwounded *Rnasel^–/–^* tissue at baseline, suggesting that these mice are primed to regenerate more robustly (Figure 2E). Corroborating previous findings that retinoic acid (RA) and TLR3 activation are required for regeneration and WIHN (9, 10), we found elevated RA levels in *Rnasel^–/–^* mice, as measured by mass spectrometry (Supplemental Figure 6, A–C). We also observed an increase in TLR3 agonist U1 snRNA (9, 11, 28) in unwounded and healed wounds of *Rnasel^–/–^* mice (Supplemental Figure 7A). These results demonstrate that RNase L loss-of-function has a proregenerative effect, seen via increased numbers of regenerated hair follicles, hair morphogenesis, and stem cell markers.

### RNase L loss increases neutrophil accumulation and IL-1 production.

**Figure 3:**
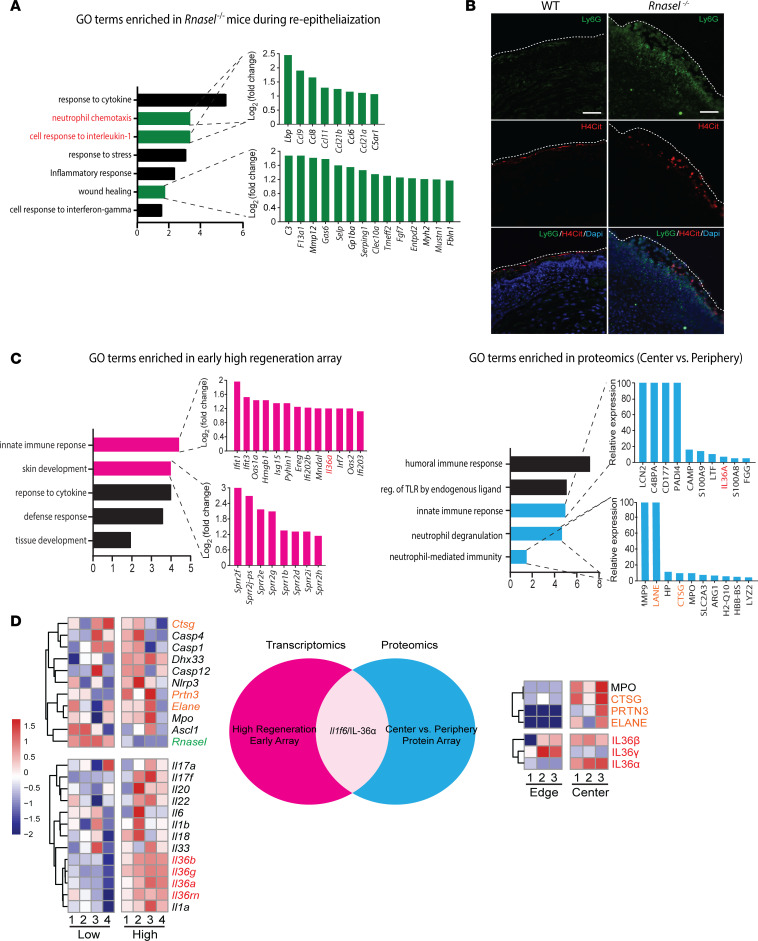
RNase L loss increases neutrophil accumulation and IL-1 production during epithelial regeneration. (**A**) Gene ontology analysis of *Rnasel^–/–^* mice during reepithelialization (approximately 10 days after wounding) show enrichment of IL-1 response, neutrophils, and wound healing pathways. Individual genes corresponding to each category are shown in green. (**B**) At 3 days after wounding, *Rnasel^–/–^* mice recruit significantly more neutrophils in the wound bed. Ly6G (green) is a neutrophil marker and H4Cit (red) is a marker for citrullinated histones released from neutrophils during NETosis. Nuclear staining was performed using DAPI (blue). The white dashed line signifies the dorsal edge of the wound bed. Scale bar: 100 μm. (**C**) Gene ontology analysis of the top 100 genes in a microarray of high regenerating outbred WT strain mice (C57BL/6 × FVB × SJL) compared with the lower regenerating WT C57BL/6 and the top 100 proteins found in the center (high regenerating) versus the edge (low regenerating) areas of the wound show a common signature for IL-1 family member IL-36α (red) and neutrophil granule proteins (orange). (**D**) Heat map analyses from **C** show IL-1 family members are enriched in the high regeneration mice and center of the wound, particularly IL-36 family members (red). Neutrophil granule proteins (orange), known to proteolytically cleave and activate IL-336 proteins, are also enriched.

We next focused on defining pathways that could explain the elevated regeneration in *Rnasel^–/–^* mice. Gene ontology analysis of the wound reepithelialization phase of WIHN in *Rnasel^–/–^* mice demonstrates an enrichment of neutrophil chemotaxis (Figure 3A and Supplemental Figure 8A). Indeed, after wounding, *Rnasel^–/–^* mice recruit significantly more neutrophils to the wound bed than strain-matched controls (Figure 3B) (29). Gene ontology analysis also reveals elevated IL-1 pathways in *Rnasel^–/–^* mice, suggesting that acute inflammation may promote regeneration (Figure 3B), consistent with published findings that IL-1 signaling promotes WIHN. (8)

To search for a candidate that explained the high WIHN in RNase L–KO mice, we next analyzed 2 scenarios of high WIHN in WT mice. A hallmark of WIHN is the topographical affinity for de novo hair follicles to form in the center rather than the periphery of the wound in mice and rabbits. (3, 4) We determined the top 100 proteins present in the center compared with the edge of the wound by proteomics (9). For the second, we determined the top 100 transcripts expressed in healed wounds of outbred mice with high WIHN (C57BL/6J × FVB × SJL) compared with a pure inbred C57BL/6J strain with low WIHN, as mentioned above (4, 10). In this intersection, we identified the specific IL-1 cytokine family member IL-36, neutrophil biological processes, and enzymes such as elastase (ELANE) (Figure 3, C and D). IL-36α is a DAMP that regulates epidermal inflammation in humans and has been seen to promote WIHN in mice (30). Genetic defects leading to gain-of-function of IL-36 are associated with neutrophilic infiltration in the skin (31–34). Distinct to other IL-1 family members that are catalytically activated by caspases, the IL-36 family (α, β, γ, and the receptor antagonist, rn) is activated by neutrophil-derived proteases like cathepsin G (CTSG) and ELANE (32, 35, 36). This suggests IL-36 as a candidate to explain high WIHN in RNase L–KO mice.

The elevation of skin-infiltrating neutrophils in RNase L–KO mice during high WIHN (depicted in Figure 3, A and B) aligns with known elevation of skin-infiltrating neutrophils in IL-36 gain-of-function models (37). Bioinformatic analysis demonstrates a correlation of IL-36 with high WIHN, and the presence of enzymes such as ELANE, known to activate IL-36. Therefore we hypothesized that elevated IL-36, specifically IL-36α, could be a downstream effect of Rnase L loss of function, potentially accounting for the elevated levels of WIHN in *Rnasel^–/–^* mice.

### IL-36α is required for WIHN and is increased in Rnasel^–/–^ mice.

**Figure 4:**
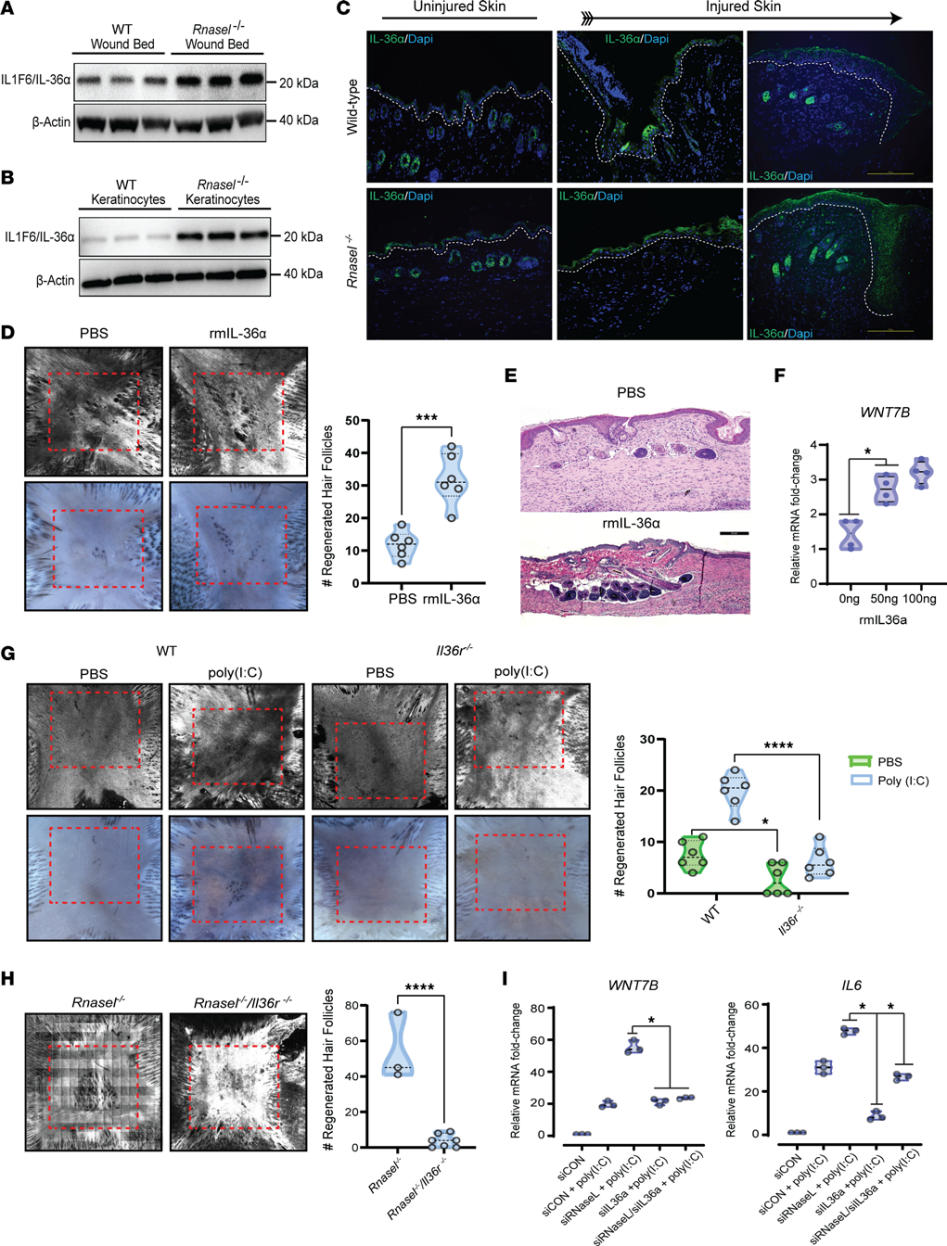
RNase L suppresses IL-36 expression, which is required for and promotes WIHN. (**A**) Wounded tissue from *Rnasel^–/–^* mice reveal elevated ILF16/IL-36α protein, as shown by Western blot. (**B**) Keratinocytes harvested and cultured from *Rnasel^–/–^* mice actively secrete more IL-36α than WT controls, as shown by Western blot. (**C**) Both unwounded and wounded skin show increased expression of IL-36α (green) in *Rnasel^–/–^* mice. IL-36α expression peaks in both WT and *Rnasel^–/–^* mice at 3 days after wounding. Scale bar: 200 μm. (**D**) Injection of 50 ng of recombinant IL-36α protein underneath the scab at WD7 promotes WIHN, as shown by CSLM and AP staining (*n* = 6, 2-tailed unpaired *t* test, *P* = 0.0002). (**E**). Histology of **D** comparing vehicle or rmIL-36α–treated mouse skin sections. The neogenic hair follicles (purple) are shown aggregated at the center of the scar. Scale bar: 200 μm. (**F**) Treatment of human keratinocytes with recombinant IL-36α increases *WNT7B* mRNA expression (*n* = 4,1-way ANOVA, *P* < 0.05), as quantified by qRT-PCR. (**G**) *Il36r^–/–^* mice fail to regenerate hair follicles and are not responsive to poly (I:C), as shown by CSLM and AP staining (*n* = 6, 2-way ANOVA, *P* = 0.0147, *P* < 0.0001). (**H**) *Rnasel^–/–^*/*Il36r^–/–^* mice lose the ability to regenerate hair follicles compared with *Rnasel^–/–^* mice, as shown by CSLM (*n* = 3 and 7, respectively, 2-tailed unpaired *t* test, *P* < 0.0001). (**I**) siRNA Knockdown of IL-36α and RNase L in human keratinocytes abrogates the increases of both *WNT7B* and *IL6* morphogenesis markers compared with RNase L siRNA alone (*n* = 3 each, 1-way ANOVA, *P* < 0.05).

To test whether IL-36 mediates the high WIHN of RNase L–null mice, we investigated the role of IL-36 in WT, *Il36r^–/–^,* and *Rnasel^–/–^* mice. After skin injury, we find that *Rnasel^–/–^* mice had more IL-36α in the wound bed (Figure 4A), and cultured *Rnasel^–/–^* keratinocytes secreted elevated levels of total IL-36α (Figure 4B). The presence of higher levels of IL-36α was also confirmed by IHC in *Rnasel^–/–^* wound beds and seen to be heightened in the epithelial lip/leading edge of inwardly migrating keratinocytes (Figure 4C), suggesting its role in healing. We then tested the effect of injected recombinant IL-36α (rmIL-36α) protein on WIHN. Compared with vehicle-treated, strain-matched controls, mice treated with rmIL-36α had more than a 2-fold increase in the number of neogenic hair follicles (Figure 4, D and E), and rmIL-36α promoted WNT7B expression in human keratinocytes (Figure 4F). Given the sufficiency of IL-36 to promote WIHN, we tested the functional requirement for IL-36 by wounding mice lacking the receptor for IL-36 (*Il36r^–/–^* mice). IL-36 receptor loss nearly abolished regeneration in vehicle-treated, strain-matched controls, which had previously shown enhanced WIHN by exogenous dsRNA (Figure 4G). To define epistasis between RNase L and IL-36, we generated and wounded a double-KO strain (*Rnasel^–/–^/Il36r^–/–^* mice). *Rnasel^–/–^/Il36r^–/–^* mice lost the enhanced regenerative capacity seen in *Rnasel^–/–^* mice (Figure 4H), suggesting that increased IL-36 activity is downstream of RNase L loss. Recapitulating these findings in vitro, simultaneous transcriptional silencing of both RNase L and IL-36α in human keratinocytes resulted in decreased *WNT7B* and *IL-6* compared with keratinocytes only targeted for RNase L (Figure 4I). These results suggest that RNase L loss leads to increased IL-36α production, enhancing WIHN. We next sought to define the mechanism by which IL-36α is induced in response to RNase L deletion.

### Caspases restrict IL-36 and WIHN.

**Figure 5:**
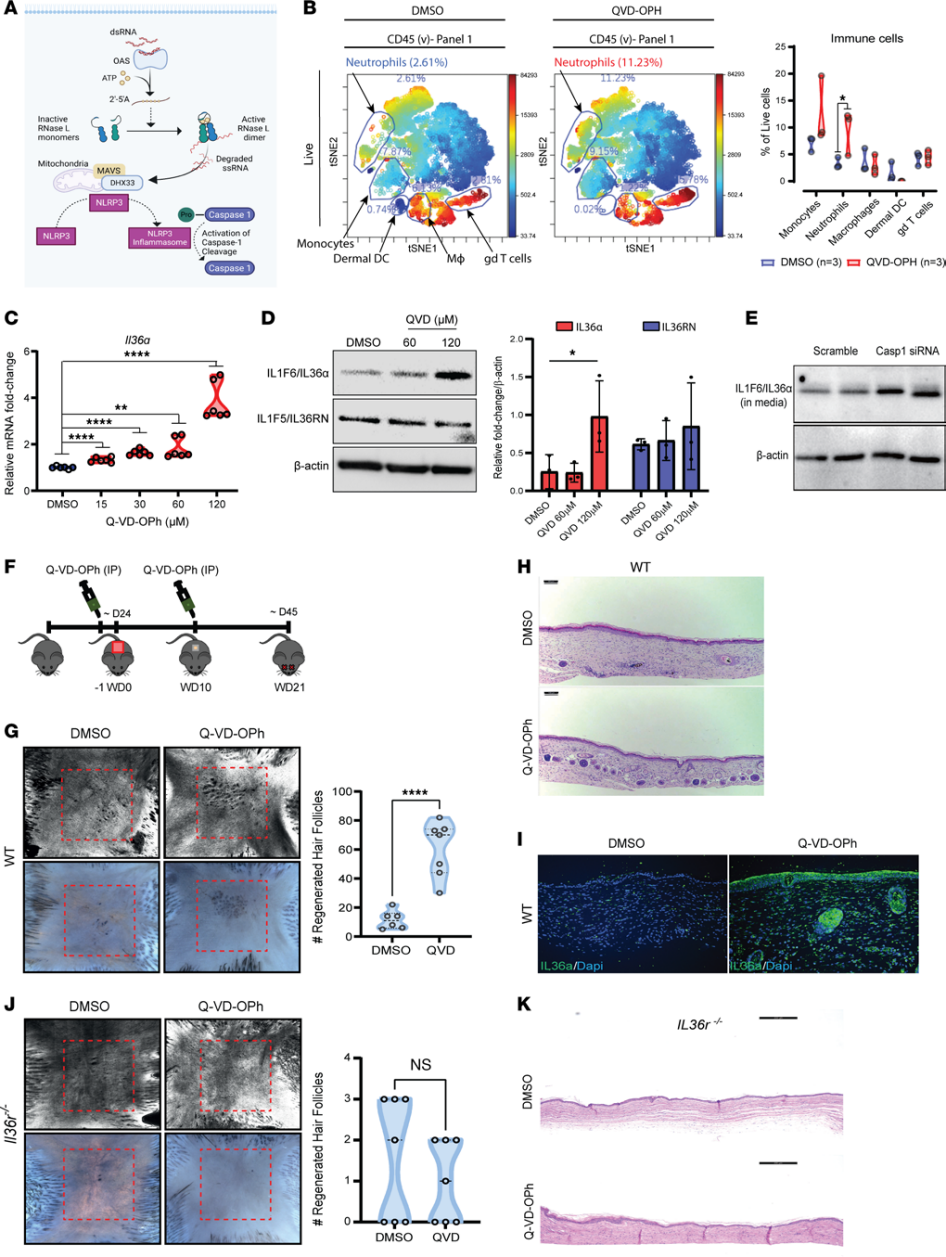
Caspases, known downstream mediators of RNase L, restrain IL-36 release and regeneration. (**A**) dsRNA activation of RNase L stimulates NLRP3 & Caspase-1 illustration. (**B**) QV-D-OPh induces elevation of neutrophils in unwounded skin shown by t-SNE analysis and quantification (*n* = 3, 2-way ANOVA, *P* < 0.05) (**C**) QV-D-OPh for 48 hours induces *Il36a* mRNA in cultured mouse keratinocytes (*n* = 6, 1-way ANOVA, ****P < 0.0001, **P = 0.0012). (**D**) QV-D-OPh induces IL-36α protein without inhibition of the receptor antagonist IL-36RN in whole-cell lysates of MEKs (*P* < 0.05 by 2-way ANOVA, *n* = 3). (**E**) siRNA knockdown of caspase-1 in mouse keratinocytes leads to elevated secretion of IL-36α. (**F**) Schematic of the pan-caspase inhibitor Q-VD-OPh intraperitoneal injection (1.33 mM) of mice wounded to measure WIHN. IP injections were done 1 day before and 10 days after wounding C57BL/6J mice with 1.25 × 1.25cm^2^ square wounds. Mice were then sacrificed at wound-day 21 to measure WIHN. (**G**) QV-D-OPh promoted WIHN compared with vehicle, as shown by CSLM and AP staining (*n* = 6 versus 7, 2-tailed unpaired *t* test, *P* < 0.0001). (**H**) Histology of **G** comparing vehicle to Q-VD-OPh–treated mouse skin sections. Neogenic hair follicles (purple) are shown aggregated at the center of the scar. Scale bar: 100 μm. (**I**) Immunostaining of reepithelialized wounded tissue shows elevated IL-36α in Q-VD-OPh–treated mice. (**J**) *Il36r^–/–^* mice do not respond to Q-VD-OPh treatment and are unable to regenerate hair follicles after wounding (*n* = 7, 2-tailed unpaired *t* test). (**K**) Histology of WIHN scars from **J**. Scale bar: 200 μm.

To define how RNase L loss leads to more IL-36α, we focused on downstream pathways with known stimulation upon RNase L activation. RNase L excites host immune defense through the activation of the NLRP3 inflammasome and subsequent stimulation of caspase-1 protease (summarized in Figure 5A) (21). We thus wondered if increased IL-36α and WIHN through RNase L loss occurred via the inflammasome. We first tested if Nlrp3 loss would increase regeneration, as occurs with RNase L knockout. Indeed, *Nlpr3^–/–^* mice had increased WIHN (Supplemental Figure 9A). Similarly, cells treated with the inflammasome inhibitor MCC950 also had greater regeneration markers than controls, which were further increased by exogenous dsRNA addition (Supplemental Figure 9B). This suggested that inflammasome downstream caspase function might be similarly required for RNase L function. We performed a small screening experiment with siRNA to multiple caspases as well as miscellaneous candidates in dsRNA-treated keratinocytes and noted caspase-1 siRNA had the greatest capacity to promote IL-36 expression (Supplemental Figure 10A).

While the suggestion that caspase-1 siRNA would promote IL-36 expression is logical given its canonical role downstream of RNase L, this was surprising, since caspases proteolytically activate cytokines such as IL-1β,which promote WIHN (8); an alternative prediction would be that caspase inhibition should inhibit WIHN rather than promote it, as suggested by the effects of RNase L/Nlpr3 loss-of-function and the aforementioned screen. Interestingly, previous reports indicate that there are decreased levels of caspase-1 (pro and activated) in stimulated Rnase L–null mice (21, 38). To confirm the above screen, we used a small molecule caspase inhibitor, Q-VD-OPh, an irreversible pan-caspase inhibitor currently in clinical testing for a number of indications, and intraperitoneally injected unwounded mouse skin. Following treatment and FACS analysis, visualization of t-distributed stochastic neighbor embedding (viSNE) clustering revealed higher neutrophil levels in the skin of mice treated with Q-VD-OPh compared with vehicle controls, reminiscent of results from *Rnasel^–/–^* mice (Figure 5B). Mouse epithelial keratinocytes (MEKs) treated with Q-VD-OPh & Caspase-1 inhibition also had higher levels of IL-36α mRNA and protein (Figure 5, C–E). No differences were seen in the protein or mRNA levels of the IL-36 receptor antagonist (IL-36RN), suggesting that elevated IL-36α levels upon caspase inhibition were not being mediated via a feedback loop with the antagonist (Figure 5D and Supplemental Figure 10B). Elevated morphogenesis genes (*Tlr3*), as seen in *Rnasel^–/–^* mice, were again detected, this time just with caspase inhibition, as were the compensatory mRNA increases of caspase genes (Supplemental Figure 10B). Increased IL-36α due to small molecule caspase inhibition was also confirmed via Z-WEHD-FMK, a Group 1 caspase inhibitor that primarily targets caspase 1 (Supplemental Figure 10C), as well as the pan-caspase inhibitor Emricasan (Supplemental Figure 10D) (39). These results therefore confirmed a likely role of caspase-1 to restrain regeneration, as is the case for RNase L.

To directly test if caspase inhibition may be equivalent to RNase L inhibition in WIHN promotion, we intraperitoneally injected mice with Q-VD-OPh (Figure 5F) 24 hours before wounding and approximately 10 days after (scab detachment). WT mice treated with Q-VD-OPh displayed more WIHN compared with vehicle-treated mice, in addition to higher levels of IL-36α in the epidermis of reepithelialized wounds (Figure 5, G–I), while *Il36r^–/–^* mice treated with Q-VD-OPh did not (Figure 5, J and K). To determine whether this increase in regeneration was skin specific, these findings were recapitulated with small molecule caspase inhibition in a dextran sulfate sodium–induced (DSS-induced) colitis model of WT and *Il36r^–/–^* mice (Supplemental Figure 11A). We observed increased IL-36α expression, reduced epithelial damage, and rescued weight loss in the gut of WT mice treated with Q-VD-OPh following gut injury. In contrast, Q-VD-OPh treatment showed no improvement in Il36r^–/–^ mice (Supplemental Figure 11, B-G). These results collectively demonstrate that caspase inhibition promotes regeneration in an IL-36 dependent manner, suggesting its potential clinical use in injury repair.

### Caspase-1 cleavage of Ticam-1 restricts IL-36α activation.

**Figure 6:**
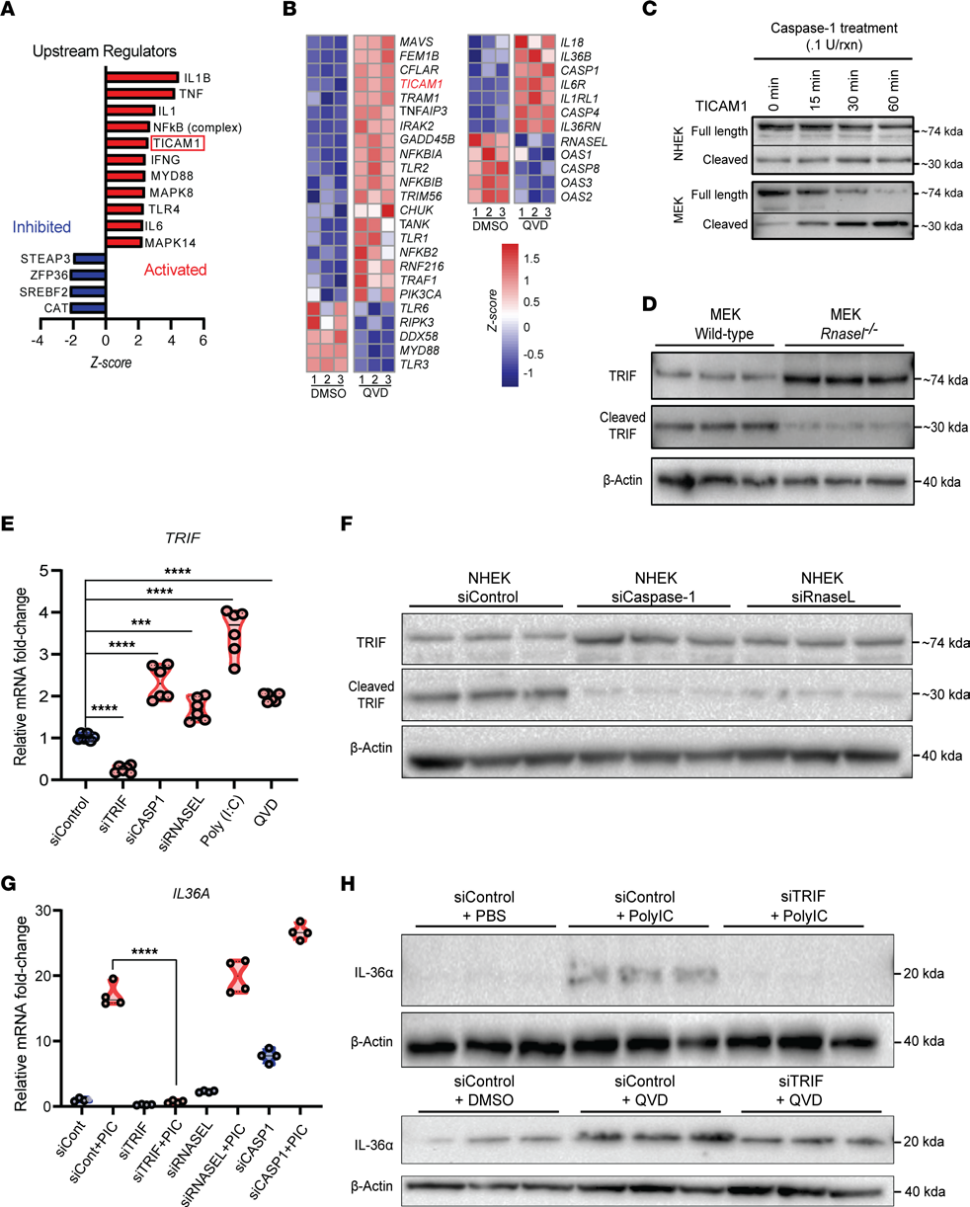
Caspase-1 cleaves Ticam 1 to restrict IL-36α and regeneration. (**A**) Based on proteome of NHEKs treated with QVD (*n* = 3) versus DMSO (*n* = 3), upstream regulators are predicted to include elevated TICAM1 (TRIF) by Ingenuity analysis (**B**) RNA-seq transcriptome of cells treated as in **A** also shows elevation of *TICAM1* (rows represent independent samples, color scale based on *Z*-score distribution) (**C**) Time-dependent cleavage of TICAM1 in unstimulated MEK and NHEK lysates after addition of recombinant caspase-1 protein and visualized by Western blot. (**D**) Western blot analysis of unstimulated *Rnasel^–/–^* MEKs show increased TRIF and decreased cleavage. (**E**) qRT-PCR quantification of *TRIF* mRNA after CASP1 and RNASEL siRNA treatment as well as poly I:C & QVD in NHEKs (*n* = 6, 1-way ANOVA, *P* < 0.001) (**F**) Western blot analysis shows Caspase-1 and RNase L-dependent TRIF cleavage after siRNA treatment in NHEKs (**G**) qRT-PCR detects *TRIF*-dependent *IL-3-6A* expression after CASP1, RNASEL, or TRIF siRNA in NHEK with or without poly I:C addition (*n* = 4, 2-way ANOVA, *P* < 0.001) (**H**) Western blot analysis of IL-36α shows TRIF-dependent protein expression with Poly I:C addition (top) or QVD (bottom) (*n* = 3).

Given that dsRNA signaling promotes innate immunity and regeneration, how inhibition of the RNase L/NRLP3/Caspase axis leads to increased IL-36α and WIHN is an important question. To gain insight into the potential mechanisms that are upregulated during caspase inhibition, we performed proteomic and gene expression analysis on normal human epidermal keratinocytes (NHEK) treated with either Q-VD-Oph or DMSO vehicle control. Consistent with the ability of Q-VD-Oph (Figure 5G) and IL-1β (8) to promote WIHN, upstream ingenuity analysis demonstrated that the IL-1β pathway was upregulated, confirming the validity of the assay (Figure 6A). Interestingly, we observed an upregulation of the TIR domain containing adaptor molecule 1 (TICAM1 or TRIF) pathway as well as its downstream activators in both proteomic and RNA-seq analysis. (Figure 6, A and B). Previous studies have shown caspase-1 cleavage of TRIF in humans as a regulator of innate immune response and autophagy (40–42) as well as TRIF deficiency resulting in decreased cytokine production and wound closure. (43, 44) We therefore hypothesized that caspase-1 might normally cleave TRIF in keratinocytes to restrain innate immunity, and caspase inhibition would inhibit this negative feedback to unfetter TLR signaling and promote WIHN.

To directly visualize potential cleavage of TRIF by caspase-1, we incubated human and murine recombinant caspase-1 with NHEK and MEK lysates. Indeed, the addition of recombinant caspase-1 reduced full-length TRIF while increasing a known cleavage fragment (40, 41) in human and mouse samples (Figure 6C). *Rnasel^–/–^* MEKs cultured from mouse back skin revealed less cleavage and higher full length TRIF compared with WT mouse skin (Figure 6D). To measure if TRIF might also be modulated at the transcriptional level, we measured *TRIF* mRNA by qRT-PCR. While TRIF siRNA decreased, QVD, Poly I:C, Caspase-1 siRNA and RNase L siRNA increased *TRIF* mRNA (Figure 6E). As expected, RNase L and Caspase-1 knockdown both resulted in a reduction in TRIF cleavage product levels, as well as a slight increase in full-length TRIF (Figure 6F), indicating that RNase L activation of Caspase-1 does promote the cleavage of TRIF. We then measured IL-36α mRNA expression in NHEKs, which showed decreased expression of *IL-36A* mRNA after TRIF siRNA treatment (Figure 6G). Inhibition of TRIF appeared to negate the stimulatory effect of Poly I:C and QVD on IL-36α expression and production (Figure 6H). Taken together, these experiments reveal that caspase cleavage of TRIF functions as a negative feedback to restrict immune stimulation and regeneration.

### ScRNA-seq and spatial transcriptomics reveal the geographic distribution of elevated OAS and IL-36 expression within wound beds.

**Figure 7:**
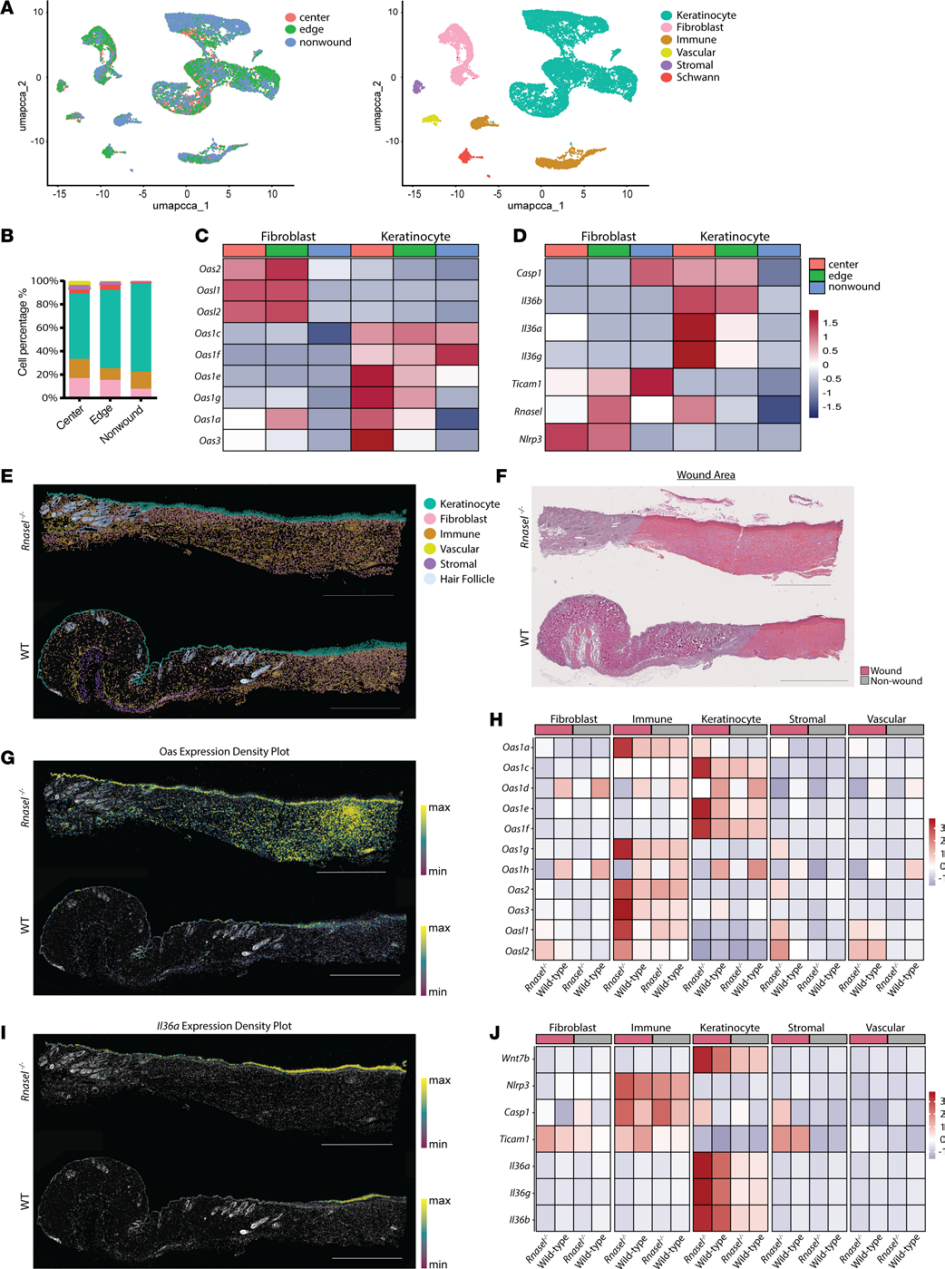
Comparative analysis of ScRNA-seq in wound center, wound edge, and nonwound regions and spatial transcriptomics in WT and RNase L–deficient models. (**A**) UMAP of WT wounding dataset showing sample integration and annotation. Clusters are color coded. (**B**) Stacked bar plot of relative fractions of each cell type. Colors indicate annotated clusters from **A**. (**C**) Heatmap comparison of OAS gene expression in keratinocytes versus fibroblasts shows higher levels of OAS expression in wound samples. (**D**) Heatmap comparison of genes of interest in keratinocytes versus fibroblasts, show higher levels of IL-36 expression in the center of wound samples in keratinocytes. For **C** and **D**, sample indicated by color, rows represent independent samples, heatmap color scale based on *Z*-score distribution. (**E**) Graph-based clustering of *Rnasel^–/–^* versus WT wound at SD0; each color-labeled dot corresponds to particular location in the tissue section (Scale bar: 1,000 μm) (**F**) Designated wound area shown on postxenium histology slide; wound area shown in pink, nonwound area shown in grey (Scale bar: 1,000 μm) (**G**) Density map of OAS transcripts in *Rnasel^–/–^* versus WT wound tissue at SD0; color gradient indicates density of transcripts per square (density threshold, 0.050; Scale bar: 1,000 μm) (**H**) Heatmap comparison of OAS gene expression from merged spatial *Rnasel^–/–^* versus WT wounding dataset (*n* = 3 and 3, respectively), shows higher levels of OAS expression in *Rnasel^–/–^* wounds (rows represent independent samples (*n* = 3) from wound area designations, color scale based on *Z*-score distribution). (**I**) Density map of *Il36a* transcripts in *Rnasel^–/–^* versus WT wound tissue at SD0; color gradient indicates density of transcripts per square (density threshold, 0.050; Scale bar: 1,000 μm) (**J**) Heatmap comparison of genes of interest expression from merged spatial *Rnasel^–/–^* versus WT wounding dataset (*n* = 3 and 3, respectively), shows highest levels of Il36 expression in *Rnasel^–/–^* wound keratinocytes (rows represent independent samples (*n* = 3) from wound area designations, color scale based on *Z*-score distribution).

Using single cell RNA-seq (scRNA-seq) and spatial transcriptomics, we aimed elucidate the cellular and topographic expression of previous results within WT and *Rnasel^–/–^* wounds. For scRNA-seq analysis, we utilized a previously generated regional wounding data set, which included skin samples from the center, edge, and nonwounded peripheral skin of WT (C57BL/6J) wounded mice at scab detatchment 0 (SD0). (45) Using CCA integration, we clustered the cells in the wound center (WIHN area), wound edge, and nonwounded skin (Figure 7A and Supplemental Figure 12A) by defined marker and DEG analysis to highlight 6 distinct cell types: keratinocyte, fibroblast, immune cells, Schwann cells, endothelial cells, and vascular cells (Figure 7B and Supplemental Figure 12, B and D). To compare expression in the epidermal (keratinocytes) and dermal (fibroblasts) layers, we generated heatmap comparisons of OAS expression across wounding regions, revealing a clear increase in *Oas1e*, *Oas1g*, *Oas1a*, and *Oas3* expression in keratinocytes at the center of the wound and increased expression of *Oas2*, *Oasl1*, and *Oasl2* in both wound center and edge fibroblasts (Figure 7C). Additional heatmap comparisons of regional gene expression showed highest levels of *Il36a* (*Il1f6*) expression in wound-center keratinocytes (Figure 7D). Wnt genes, known drivers of WIHN (4), and IL1 signaling pathways were also shown to be higher in center wound keratinocytes (Supplemental Figure 12, E and F), while *Il36r* (*Il1rl2*) showed higher expression in fibroblasts. A previously generated Visium spatial transcriptomic dataset (46) comparing unwounded and wounded (POD 14) skin confirmed that distribution of *Il36a* was highest in wounded epidermal skin layer, and that Rnase L & OAS genes are higher but expressed in both the epidermis and dermis of POD14 wounded skin (Supplemental Figure 13, A and B).

To further investigate the topologic distribution of these genes and their influence on different cell types, we probed the spatial transcriptomics of *Rnasel^–/–^* versus WT (C57BL/6J) mouse wounds collected at SD0 (*n* = 3 and 3, respectively). We created a custom probe set based on dsRNA sensing, regeneration, and other immunologically relevant pathways, as well as the mouse tissue atlas platform from 10X Genomics(probes are listed in Supplemental Table 5). All samples were merged and clustered using Leiden algorithm in Giotto (47) (Supplemental Figure 14A), and visualized in Xenium Explorer (Figure 7E and Supplemental Figure 14, C and E). Wound areas were determined using post-xenium histology slides, which were then imported and aligned into Xenium explorer, with cells in the wound areas highlighted (Figure 7F and Supplemental Figure 14, D and F). *Rnasel^–/–^* mice expressed higher levels of OAS transcripts across the epidermis and dermis compared with WT controls (Figure 7G). In particular, *Oas1c*, *Oas1e*, and *Oas1f* were most elevated in *Rnasel^–/–^* wounded keratinocytes and *Oas1a*, *Oas1g*, *Oas2*, *Oas3*, and*Oasl1* were most elevated in *Rnasel^–/–^* wounded immune cells (Figure 7H). Similarly, *Rnasel^–/–^* mice expressed more *Il36a* in the epidermis of wounded skin compared with WT mice (Figure 7I). Heatmap comparisons confirmed that *Il36a* was highest in *Rnasel^–/–^* wounded keratinocytes, along with morphogenic markers (Figure 7J and Supplemental Figure 14B). Taken together, these data demonstrate that the elevated expression of OASs and *Il36a* in *Rnasel^–/–^* wounds correlate with elevated regeneration.

## Discussion

In this study, we have identified RNase L as a novel repressor of WIHN, thereby establishing a new role for antiviral innate immunity in the process of regeneration. Additionally, we have pinpointed IL-36α as a crucial marker of regeneration and demonstrated its restraint via RNase L, NLRP3, and subsequent Caspase cleavage of TRIF. Our results are consistent with the hypothesis that limited regeneration in humans and other mammals is an evolutionary adaptation, possibly to restrict excessive inflammation in immunity and carcinogenesis (48), but at the cost of inhibiting regenerative responses.

Following tissue damage, immune cell activation and inflammation at the site of injury play an important role during regeneration. Recruitment of Ly6C^+^ inflammatory macrophages (7) and γδ T cells in damaged tissue can regulate Lgr5^+^ hair follicle stem cell activation and epithelial proliferation (6). The release of cytokines is a key factor in the regeneration of hair follicles, with their inhibition resulting in decreased WIHN (8). A logical predication, given the promotion of WIHN by dsRNA and Tlr3 (10) is that the restraint of other dsRNA sensors and their subsequent pathways (49) would yield similar results. We surprisingly found that, while OAS levels universally correlated to high regeneration, downstream activation of RNase L acted as a regeneration repressor, decreasing WIHN. This revealed a paradoxical function of the innate immune system, where dsRNA sensing functions in both an inflammatory and an antiinflammatory capacity. The spatial distribution and increased expression of OAS genes in the center of wounds and in *Rnasel^–/–^* wound beds raises the important question of what the RNaseL-independent proregenerative effects of OAS are. Whether this is cell-cycle modulation by OAS genes, perhaps during the cellular stress of wounding, or IFN-related modulation by OASs, remains to be further investigated. We also find that during wounding, caspases perform physiological functions beyond their established role in IL-1 activation during cellular stress events (8, 50), acting as an antiinflammatory agent via the restraint of TLR signaling. This increased capacity for regeneration and immune recruitment following caspase inhibition highlights the role of caspases as regulators of regeneration (51) and immune response tuning.

IL-36 is a multifunctional cytokine with an array of biological functions such as immune cell differentiation, metabolism regulation, and tissue regeneration (30, 31, 33, 52). Similar to previously published results (30), we found IL-36α to be a powerful stimulant of WIHN. Single-cell and spatial data clearly demonstrate a significant increase of IL-36α in wound beds, with highest levels observed in keratinocytes. Wounding and colitis experiments in *IL-36r^–/–^* mice as well as Q-VD-OPh’s inability to stimulate epithelial regeneration in *IL-36r^–/–^* mice reveal that IL-36 not only stimulated WIHN but was critical for a generalized epithelial healing response. Our study shows that caspase-1 inhibition had a clear correlation with IL-36α via both small molecule inhibition and targeted siRNA knockdowns. Analysis of IL-36α in Q-VD-OPh–simulated NHEKs after TRIF knockdown reveals only a partial reduction in IL-36α. This is consistent with partial siRNA efficacy, as well as alternative IL-36α promotion mechanisms, such as Q-VD-OPh’s likely inhibition of caspase cleavage of MyD88 (53), an alternative signaling pathway to TRIF acting on most TLRs and IL-36R. Supporting our work, treatment with Q-VD-Oph has also been shown to stimulate IFN-β expression, similar to RNase L KO, and increase p-IRF3, known to be downstream of the dsRNA-TRIF pathway. (54)

While this study suggests an epidermal role of IL-36α in wound healing, our study has several limitations. The lack of functional assays in a keratinocyte-specific IL-36α–knockout model restricts our ability to fully attribute the effects observed in the wound microenvironment to keratinocyte-derived IL-36α. Future work utilizing conditional-knockout mice with cell type–specific deletion of IL-36α and or Rnase L will be crucial to pinpoint their precise roles in wound healing. Additionally, tracking the spatiotemporal expression and interactions of these signaling components in vivo could provide valuable mechanistic insights into their contribution during the healing process. Future work reflecting these considerations will help refine and strengthen the model proposed here, allowing for a more comprehensive understanding of IL-36α’s function in skin wound repair.

Another key question is how immunoregulatory pathways, such as the RNase L/NRLP3/Caspase axis, contribute to the differences in skin thickness and durability between volar (nonhair-bearing) and nonvolar (hair-bearing) regions. Previous work from our lab demonstrated that dsRNA sensing via DExD/H-Box Helicase 58 (DDX58) helps maintain keratinocyte stemness and skin integrity by promoting KRT7 expression and inhibiting KRT9, a marker of volar differentiation. (55) Results from our xenium data further corroborate this, revealing elevated *Ddx58* levels in the dermis and increased *Krt7* expression in keratinocytes within high regenerating RNase L knockout wounds. Additionally, RNase L–KO mice exhibit reduced hypodermal thickness compared with WT mice, consistent with an association of KRT9 with hyperkeratosis. (56) This suggests that the RNase L/NRLP3/Caspase axis promotes skin barrier integrity and resilience in both volar and nonvolar areas, inhibiting follicular regeneration in hair-bearing regions, where morphogenesis and tissue regeneration are more active. This regulation of dsRNA sensing suggests how immune signaling might contribute to the regional specialization of skin, both in normal physiology and wound healing contexts.

Highlighting the clinical relevance of the surprising antiinflammatory nature of this arm of antiviral innate immune signaling is a recent report on gene susceptibilities to hyperinflammation after COVID. MIS-C (22) is a syndrome of excessive inflammation after COVID, which has recently been attributed to inherited mutations in the OAS/RNase L pathway. Therefore, the cleavage of TRIF and MyD88 by caspase-1 illustrates a biologically coherent mechanism to limit inflammation in multiple contexts. Future work should demonstrate other physiologically relevant instances of caspases to limit inflammation, as well as how IL-36α recognition stimulates WIHN and tissue regeneration.

Our study uncovers a novel role for the OAS and RNase L pathway within tissue regeneration. Although RNase L is known to have important functions outside of classic antiviral immunity, such as in prostate cancer (57), it has not been connected to developmental pathways or stem cell behavior. We find that RNase L physiologically restricts the activity of wound-activated stem cells to promote regeneration. It is well appreciated that hyperactivated antiviral immune responses in humans can lead to significant morbidity and mortality, such as during SARS-CoV2 infection. In this context, it is intuitive for virally activated pathways, such as RNase L, to have parallel antiinflammatory negative feedback loops to fine tune immune responses. Our work suggests that this tradeoff also limits regenerative responses after injury.

In summary, we demonstrate an important paradox where dsRNA promotes WIHN, but the dsRNA-activated enzyme RNase L inhibits WIHN, through caspase cleavage of TRIF and inhibition of IL-36α. These findings suggest that the inhibition of caspases and RNase L might have therapeutic benefit to epithelial injury and may be useful in humans after acute injuries such as skin burns or bowel perforation.

## Methods

### Sex as a biological variant.

Our study examined male and female animals, and similar findings are reported for both sexes; therefore, sex was not considered as a biological variable.

For additional methods, please see Supplemental Methods.

### Mouse lines.

All WT and control mice used for in vivo experiments were on the C57BL/6J background. All mice were age-matched and cohoused until 6-weeks of age. *Rnasel*-KO mice (*Rnasel*^tm1Slvm^) (18), provided in-house, were previously backcrossed on a C57BL/6J background. The *Il36r*-KO mice (*Il1rl2^tm1Hblu^*) were acquired through a material transfer agreement between Johns Hopkins University School of Medicine and Amgen, Inc. Rnase L and Il36r–double-KO mice were generated by crossing both strains until viable homozygous mice were produced. All transgenic variants used were genotyped to confirm transgenic status using corresponding primers (Supplemental Table 1).

### WIHN assay.

All in vivo experimental surgical procedures were performed as previously characterized (4–6, 9,10). In short, after exposure to vaporizing anesthesia (Baxter, Isoflurane) the dorsal side of male and female 3-week-old (21 days) mice, approximately 8–11g of weight, were shaved. Using surgical scissors, approximately 1.26 cm^2^ × 1.25cm^2^ of skin was excised, creating wounds deep into the fascia. At 3 days after wounding, a single dose (50 μL) of 100 μg/mL high molecular weight (HMW) Poly (I:C) (Invivogen, tlrl-pic) was administered underneath the scab site via injection in both WT and transgenic mice, as previously described (9, 10). For functional experiments characterizing IL-36 on WIHN, a single dose (50 μL) of 1 μg/mL recombinant mouse IL-36α protein (R&D Systems, 7059-ML/CF) was injected underneath the scab site at 7 days after wounding. For rescue experiments using the small molecule, broad spectrum pan-caspase inhibitor, 20 μL of 5mg/mL Q-VD-OPh (in DMSO) was diluted 130 μL of PBS (1.33 mM final concentration) and injected intraperitoneally in mice 24 hours before wounding and approximately 10 days after wounding (scab detachment). After approximately 3 weeks after wounding (roughly 21 days), neogenic hair follicles in the reepithelialized skin tissue were quantified using Alkaline Phosphatase staining (NBT/BCIP, Roche) and or by reflectance confocal scanning laser microscopy (CSLM) as previously demonstrated (4, 9, 10, 58). All WIHN images (WIHN images file) and WIHN counts (WIHN counts file) have been provided.

### Human and mouse keratinocyte isolation and culture.

Primary human keratinocytes were isolated from fresh neonatal foreskins stored in CO_2_-independent medium (Gibco, 18045088), as previously described (9, 10). After removing subcutaneous fat, foreskin tissue was enzymatically digested overnight at 4˚C in a 0.4% dispase II solution (Sigma-Aldrich, D4693). After tissue disaggregation, the epidermis was incubated in either 0.025% trypsin/EDTA (Lonza, CC-5012) at 37˚C or accutase (CELLnTEC, CnT-Accutase-100) at room temperature (approximately 23˚C). The epidermal sheets were passed through a cell strainer to obtain keratinocytes that were cultured in keratinocyte growth medium (KGM-Gold) supplemented with necessary growth factors and antibiotics (Lonza, 192060). Primary mouse keratinocytes were isolated from tails as described above, with the addition of a 10 μM rho-kinase inhibitor (Y-27632, Cayman Chemical, 10005583) in KGM-Gold media, as demonstrated previously (59). All human and mouse keratinocytes were passaged at least once before use in all in vitro experiments. MEKs were treated for 48 hours with Z-WEHD-FMK (5 or 20 μM), Emricasan (5 or 10 μM), or DMSO vehicle control.

### In vitro supernatant protein concentration.

To measure secreted IL-36, protein isoform media supernatant from cultured mouse or human keratinocytes was concentrated using an Amicon Ultra centrifugal filter device with a nominal molecular weight limit (NMWL) of 3 kDa and 10 kDa (Sigma-Aldrich, UFC200324). All samples were concentrated approximately 20 × prior to use for downstream applications (i.e., immunoblotting).

### RNA isolation and quality analysis.

Total RNA (including small noncoding RNA) from tissue was isolated using a TRIzol-based, nonphase separation spin column purification method (Zymo Research, R2073). Total RNA from human keratinocytes was purified using the RNeasy Mini Kit (Qiagen, 74106). In both instances, RNA was incubated with DNase I to efficiently digest DNA prior to elution. RNA purity and quantity was calculated using a UV-Vis spectrophotometer (NanoDrop2000c [Thermo Fisher Scientific, ND-2000c]). Total RNA quality was assessed by measuring 28S/18S ribosomal RNA ratios and scoring RNA Quality/Integrity Number (RQN/RIN) values via capillary electrophoresis using either 2100 Bioanalyzer (Agilent) or Fragment Analyzer CE (Agilent).

### Quantitative real-time PCR.

A high-capacity reverse transcription kit (Applied Biosystems, 4368813) was used to synthesize cDNA from mRNA. TaqMan probes against target genes of interest were designed using fluorescein (FAM) dyes. Probes against housekeeping genes RPLP0 and β-actin were used for human and mouse-derived cDNA, respectively. Relative gene expression was determined using the comparative (ΔΔ*C*_t_) method, derived from cycling threshold (*C*_t_) differences from target and housekeeping genes.

### Flow cytometry.

Cell suspensions were prepared by digesting mouse skin tissue in a cocktail consisting of Liberase TL (Roche, 5401020001), DNase I (Sigma-Aldrich, DN25) and antibiotics (Invitrogen, 15140) in RPMI 1640 (Gibco, 11875093). For FACS, cells were washed and first viability stained using the Zombie Aqua dye (BioLegend, 423101). After blocking, cells were stained with an antibody cocktail (Supplemental Table 2) and BD Horizon Brilliant Violet (BV) buffer (BD, 563794) to minimize nonspecific spectral overlap of similar conjugates. Finally, cells were resuspended in BD stabilizing fixative prior to FACS. All flow cytometry experiments were performed on a BD LSR II and downstream analysis of data was performed using Cytobank.

### In vivo microarray.

Total RNA was isolated from mouse tissue at the time of skin reepithelialization and scab detachment from the wound (approximately 10 days after wounding) from both WT and *Rnasel^–/–^* mice. RNA was submitted to the JHMI Deep Sequencing & Microarray core facility and profiled using the Affymetrix Clariom S mouse array platform according to the manufacturer’s protocols. Gene chips were scanned generating CEL pixel intensity files, which were processed and analyzed using Partek Genomics Suite software and Robust Multichip Analysis (RMA) algorithm was used for normalization.

### In vitro RNA-seq.

Total RNA (including small RNAs) from primary human keratinocytes treated with nontargeting and RNase L–targeting siRNA in the presence or absence of 10 μg/mL poly(I:C), as well as primary human keratinocytes, were submitted to the Experimental and Computational Genomics Core (ECGC) at the Sidney Kimmel Comprehensive Cancer Center (SKCCC; Baltimore, Maryland, USA) for RNA-seq. Total RNA from NHEK’s treated with either 120 μM/mL of Q-VD-OPh (*n* = 3) or DMSO (*n* = 3) were submitted to Novogene for Sequencing. Libraries were prepared using the TruSeq Stranded Total RNA LT Sample Prep Kit (Illumina, 15031048) for polyadenylated RNA selection, followed by barcoding. Sequencing was performed on the HiSeq2500 platform & NovaSeq 6000 (Illumina), producing 50 million 100 × 100-bp paired-end reads. Illumina’s CASAVA 1.8.4 was used to convert BCL files to FASTQ files using default parameters. RSEM-1.3.0’s EBSeq was used for differential expression analysis and for running the alignments as well as generating gene and transcript expression levels. For QVD versus DMSO results FDR was calculated using Benjamini and Hochberg’s approach, genes with an adjusted *P* value of less-than 0.05 found by DESeq2 were assigned as differentially expressed. All data were aligned to the GRCh38 reference genome using the spliced transcripts alignment to a reference (STAR) method. RNase L siRNA sequencing uploaded data can be found at NIH GEO GSE164667. QVD versus DMSO sequencing uploaded data can be found at NIH GEO GSE214452.

### Histology.

Biopsies from mouse colon and skin tissue were removed and fixed in 4% paraformaldehyde overnight and then transferred to 70% ethanol. Samples were then submitted to the Johns Hopkins Oncology Tissue Services Core facility (Baltimore, Maryland, USA) where they were embedded in paraffin. Tissue sections were obtained at 4 μm thickness and mounted onto glass slides, followed by H&E staining.

### TRIF cleavage assay with recombinant caspase-1.

Recombinant mouse and human Caspase-1 and the protocol were provided by the Larman lab (Johns Hopkins University, Baltimore, Maryland, USA) (60). Unstimulated NHEK’s and MEK’s were lysed using M-PER lysis buffer with 200 μL of buffer per sample (approximately 1 × 10^6^ cells). Protease digests were set up with 50 μL of lysate (approximately 250,000 cells) adding 0.1 U/μL rCaspase-1. A 50 μL sample was set aside for the 0 minute time point and mixed with 17 μL of 4× NuPage LDS sample loading buffer. Master mix was set up for the 3 digest time points (150 μL lysate + 3 μL rCaspase-1). A total of 50 μL was taken at each time point and mixed with 17 μL of 4 × NuPage LDS sample loading buffer to stop the digestion. All samples were denatured by heating to 95°C for 5 minutes before being loaded on a gel for Western blotting.

### Proteomics analysis.

Keratinocytes from WT and *Rnasel^–/–^* mice, NHEK treated with either 120 μM/mL of Q-VD-OPh (*n* = 3) or vehicle DMSO, were prepared for protein analysis. Briefly, after saline washing, samples were lysed in 5% sodium deoxycholate (DOC) detergent. After sequential peptide processing (reduction, alkylation, and trypsinolyzation), downstream resolving and analysis were performed on a nanoACQUITY UPLC system with a Tribrid Orbitrap-quadrupole-linear ion trap mass spectrometer (Thermo Fisher Scientific). The UniProt mouse reference proteome was used to align tandem mass spectra (MS-MS) data in conjunction with the Sequest HT algorithm. Protein abundance ratios were calculated by comparing MS1 peptide ion intensity peaks. Machine-learning–based software (Percolator) was used for peptide identification and validated at an FDR of at least 0.05. Pathway, upstream regulators, and gene ontology analysis were conducted with Qiagen Ingenuity, Panther GO, and DAVID databases. Center versus Edge proteomics is available via the PRIDE partner repository with the dataset identifier PXD013854. The RNaseL proteomics is available via the PRIDE partner repository with the dataset identifier PXD061723. QVD vs DMSO proteomics is available via the PRIDE partner repository with the dataset identifier PXD036948.

### RNA isolation and quantitative real-time PCR.

Total RNA was isolated from cultured keratinocytes using RNeasy Mini Kit (Qiagen, 74106) and treated with DNase I (Qiagen, 79254) to eliminate genomic DNA. The purity and concentration of RNAs were analyzed using a NanoDrop2000c (Thermo Fisher Scientific, ND-2000c). Following reverse transcriptase reactions using high-capacity RNA to cDNA kit (Life Technologies), qRT-PCR was performed to measure target genes using TaqMan probes and Fast reaction master mix reagents (Life Technologies). Relative expression of mRNAs was analyzed by the cycle of threshold (Ct) value of target genes and quantified by normalizing to β-Actin using the ΔΔCt method.

### Spatial transcriptomics preparation.

WT and *Rnasel^–/–^* 3-week-old mice were wounded, approximately 1.25 × 1.25cm^2^, in parallel, as previously described; GSE278431 NCBI GEO. Wounded tissue from WT and Rnasel^–/–^ mice (*n* = 3 for each group) was collected at SD0. The samples were formalin fixed and paraffin embedded (FFPE) and submitted to the Johns Hopkins Oncology Tissue Services Core facility. Paraffin-embedded tissue sections were mounted on Xenium slides and then submitted to the Johns Hopkins Single Cell & Transcriptomics Core (Johns Hopkins Single Cell & Transcriptomics Core, Baltimore, Maryland, USA). Spatial transcriptomics was performed using the Xenium Analyzer (10x Genomics) following the manufacturer’s instructions. A custom panel consisting of 100 genes, in conjunction with the 10x mouse panel (see Supplemental Table 5), was targeted. Post-Xenium H&E staining was performed for each sample.

### RNaseL microarray analysis.

Protein and gene annotation enrichment and ontology analyses were performed using the Database for Annotation, Visualization and Integrated Discovery (DAVID) and the PANTHER classification system. All gene list exploratory analyses were statistically significant using the Fisher’s exact test, with the Benjamini–Hochberg FDR correction or Bonferroni correction. RNaseL-null mouse microarrays are available in GSE164003 NCBI GEO.

### Cross-array analysis.

GSE50418 NCBI GEO was used for outbred and inbred strain analysis; GSE131789 NCBI GEO was used for human laser array; GSE92646 NCBI GEO was used for Poly I:C–treated keratinocytes. To avoid directly comparing the signal values between different microarray platforms, instead we compared their fold change results, since the genes’ expression fold-change values themselves derive from comparing like (from the same array) values, which avoids any array-to-array idiosyncrasies. Each array experiment thus generated a ratio for each probe set of high regenerating sample over low regenerating sample. Also, defined orthologous genes allows expression results to be compared between different species and model organisms. Using these principles, we compared the previous results from human participants, using the Affymetrix PrimeView array, with those from mice using the Affymetrix mouse MoEx-1_0-st-v1 and Mouse Clariom_S arrays (9). The gene transcript annotation of each array was first updated to current HUGO/MGI/NCBI nomenclature, after which the mouse genes were mapped to their human orthologs using the NCBI HomoloGene and SMI databases, along with the Human Gene Nomenclature Committee’s HGNC Comparison of Orthology Predictions (HCOP) database (61). Among a standard gene set that was the identical for every experiment, the respective log_2_ fold changes for each orthologous gene in each of the 3 biological comparisons were then rank ordered from those with the highest average regeneration ratio to lowest regeneration ratio. That rank order was averaged across the 3 experiments and again sorted from highest to lowest average.

### Xenium spatial analysis.

Using Xenium explorer software (version 3.0.0) WT versus *Rnasel^–/–^* wound samples were visualized and aligned to their post-xenium H&E slides. Wound areas were determined via tissue histology, and wound-versus-nonwound cell IDs were then extracted. Transcript density maps were generated for genes of interest in xenium explorer software, density map bin size set to 10 μm within a map scale threshold of 0.00–0.05. All samples were then imported and merged into R using the Giotto Suite (4.0.8). The merged data set was then filtered for cells with at least 10 UMI counts per cell and normalized with a scale factor of 5,000. Highly variable features were then calculated before dimension reduction via principal component analysis (PCA), before UMAP visualization and clustering through Nearest Network and Leiden algorithm (resolution = 1.5). Clusters were then grouped and annotated using known DEGs and markers, then reintegrated back into Xenium explorer to confirm accuracy. Cell types not found in both wound and nonwound areas were not subclassed for heatmap comparison. Scaled expression values per cell type per condition were used to calculate relative z-scores and visualized using the heatmap function within the Giotto Suite. Sample files can be found at GSE278431 NCBI GEO.

### Visium spatial analysis.

Visium (10x Genomics) spatial samples (“Unwounded” and “POD14”) were downloaded from GSE178758 and reanalyzed in Python using Scanpy (version 1.10.1). Cells were filtered according to the specifications outlined in the original dataset. The samples were normalized, followed by principal component analysis (PCA) and uniform manifold approximation and projection (UMAP). Clusters were identified using the Leiden algorithm in Scanpy (resolution = 1). Cells were mapped based on the histological and marker data presented in the original paper. Relative gene expression was visualized through the Scanpy spatial function.

### scRNA-seq bioinformatic analysis.

We used a previously generated scRNA-seq data set, GSE273111, which obtained samples from the center, edge, and nonwounded skin of mice at SD0. Seurat package (5.1.0) was used for data processing and analysis. Genes not detected in at least 3 cells were discarded, and cells with more-than 200 genes and mitochondrial percentage below 25% were included in the analysis. After QC, samples (center, edge, nonwound) were normalized using log normalization and scaled before dimension reduction via principal component analysis (PCA), then integrated using CCAIntegration. Identified clusters were visualized using UMAP, clustered via Leiden algorithm (resolution =.5), then grouped and annotated using known DEGs and markers. Relative expression heatmaps for genes within each cell type across sample conditions were obtained by comparing pseudobulk expression of center, edge, and nonwound samples and generated using DittoSeq (1.16.0). Interactions between different cell types were analyzed with R package Cellchat (1.6.1) (62)

### PPI analysis.

Blastx alignment was used find the interaction relationship of genes from QvdvsDMSO RNA-seq in STRING database; interaction data was then used for each sample.

### Heatmap analysis.

Gene expression was standardized for definition of differentially expressed genes, and GO and KEGG enrichment analysis. We used the R package limma to define DEGs among different groups (63). We used the clusterProfiler R package for gene-annotation enrichment analysis (64). Finally, the visualization of these results was created using the ggplots2 & pheatmap R package.

### Statistics.

All in vivo and in vitro experiments were performed in at least individual instances. Univariate statistical analysis was performed using 2-tailed unpaired Student *t* test and multivariate analysis was performed using 1-way and 2-way ANOVAs. All statistical analyses and graphical representations were generated using GraphPad Prism software. Statistical significance is defined as *P* values < 0.05 derived from the SEM calculations.

### Study approval.

All mice were bred and housed at an American Association for the Accreditation of Laboratory Animal Care–compliant facility and all experimental procedures were reviewed and approved by the Johns Hopkins University Institutional Animal Care and Use Committee (IACUC). Primary human keratinocytes isolated from fresh neonatal foreskins, were done in compliance with Johns Hopkins University IRBs (NA_0033375, NA_00075350, IRB00028768).

### Data availability.

Data that supports the findings of this study are available within the article and its supplemental tables and figures. Sequencing data for RNA-seq and spatial sequencing has been deposited in the NCBI’s Gene Expression Omnibus (GEO GSE164667, GSE214452 and GSE278431). Please see individual methods for further information about other datasets used in this study. Values for all data points in graphs are reported in the Supporting Data Values file; all data points related to WIHN assay can be found in the WIHN counts file.

## Author contributions

CSK, EW, NI, and LAG wrote the manuscript and designed the study; coauthorship order was determined by work done on present manuscript. CSK, EW, NI, MPA, GW, ES, AL, SSL, AMO, YX, and WH helped acquiring and analyzing data. AMO wrote bioinformatic scripts for xenium data. HL, DK, GW, YX, SR, NKA, LSM, JY, JWJ, SK, MAK, and RHS contributed to scientific discussions.

## Supplementary Material

Supplemental data

Unedited blot and gel images

Supplemental tables 1-5

Supporting data values

## Version 1 02/04/2025

In-Press Preview

## Version 2 03/17/2025

Electronic publication

## Version 3 03/17/2025

Corrected panel placement error in Figure 7
