# Supplementary material for: RNase L represses hair follicle regeneration through altered innate immune signaling
Source: J Clin Invest. 2025 Feb 4;135(6):e172595. doi: 10.1172/JCI172595 (PMC11910212; doi:10.1172/JCI172595)
Supplement: Supplemental data [file jci-135-172595-s008.pdf]

S1

A

	Gene Symbol	PolyIC vs. Vehicle Lin(FC)	Gene Symbol	Photoaged arm post laser vs. Photoaged arm Lin(FC)	Gene Symbol	Mixed (High reg.) vs. C57 (low reg.) Lin(FC)
1	CXCL5	210.534	SPP1	5.52362	SPINK7	4.49863
2	CXCL11	183.556	IFI6	3.96393	IFIT1B	3.91942
3	RSAD2	123.893	OAS2	3.66137	MCAM	3.86255
4	C15orf48	98.8833	IFI44	3.59574	FIBIN	3.84663
5	CXCL10	90.308	MX1	3.25883	LCE3D	3.69138
6	BST2	49.356	IFI44L	3.0354	IFI44	3.67788
7	CCL4	44.1555	CCL18	2.97578	KERA	3.47377
8	KYNU	44.154	MMP1	2.94749	SLFN12	3.15464
9	MMP9	42.8771	IFI27	2.92123	TMPRSS11/	3.13814
10	CCL5	39.9065	SELE	2.88215	ZC3H11A	3.06934
11	SPOCK1	36.0099	OAS1	2.8558	IGFBP4	3.04636
12	CMPK2	35.0367	KRT6C	2.82079	USP18	3.0324
13	IL8	34.1301	FCGR3A	2.73452	ANGPTL1	2.96337
14	NDRG4	27.9493	FCN1	2.65527	LCE3C	2.91929
15	APOBEC3A	26.8785	CXCL9	2.63725	BLOC1S6	2.79603
16	ISG20	25.4395	CMPK2	2.61542	CNKSR1	2.76715
17	CD200	23.0198	SERPINB3	2.58424	OAS1	2.7335
18	CCL4L2	22.4899	HERC6	2.57601	KLK14	2.68324
19	IFIT2	21.9108	EPSTI1	2.48545	ISG15	2.6225
20	OASL	21.4159	LYZ	2.47504	KLK9	2.54324
21	CSF2	21.3788	IFIT1	2.46215	HIST3H2A	2.52509
22	ICAM1	21.2614	XAF1	2.46046	RGS5	2.52319
23	CTSS	19.8604	CXCL10	2.41908	MLL5	2.47878
24	BIRC3	19.4167	IFNK	2.40399	EPGN	2.45346
25	CXCL3	18.654	CXCL8	2.37968	SPRR3	2.4507
26	HCP5	18.5842	OAS3	2.18782	ADAMTS9	2.43706
27	ESM1	17.1361	RSAD2	2.17567	RPL29	2.40536
28	NRCAM	16.682	MSR1	2.17188	SYCP1	2.39774
29	TLR2	16.4533	CYBB	2.16895	TSLP	2.37984
30	IFIT3	15.4436	CD163	2.12115	EREG	2.3505
31	C3	14.2188	KRT16	2.10528	ERBB4	2.30356
32	MX2	13.9249	PARP9	2.08968	IL36A	2.30204
33	ZP4	13.714	KRT9	2.0501	IRF7	2.2975
34	USP18	13.0501	INA	2.04945	OAS2	2.28507
35	HLA-F	12.9915	RGS18	2.04684	PLA2G4D	2.2819
36	IFIT1	12.6305	MS4A6A	2.04628	PHF11	2.20454
37	IL32	12.6008	PLAC8	2.03164	IFNK	2.15433
38	CXCL1	12.413	SERPINE2	2.02391	FZD10	2.10741
39	C1R	11.9176	AKR1B10	1.9971	SLFN13	2.10702
40	SAMD9L	11.9099	OASL	1.98149	CXCL11	2.10593
41	SOD2	11.8183	ADH7	1.95523	DEFB103B	2.08947
42	IL1R2	11.5515	FPR3	1.95347	OAS3	2.07483
43	MMP10	11.4424	C3AR1	1.95249	RSAD2	2.05891
44	HLA-G	11.2765	PLA2G2A	1.94461	GJB6	2.05625
45	HSD11B1	11.0379	C1orf162	1.92605	KCNMA1	2.04681
46	APOBEC3B	10.2256	SAMSN1	1.92151	CUTC	2.01898
47	IL15	10.0685	CH25H	1.91909	OASL	1.99938
48	MRGPRX3	9.95479	ISG15	1.91766	AGTR2	1.9984
49	HSH2D	9.84545	EVI2A	1.9133	ZBP1	1.99445
50	AKAP12	9.64196	C10orf99	1.9102	GTPBP4	1.99275
51	DDX58	9.09833	PTPRC	1.89247	C2orf40	1.984
52	SAMD9	9.08144	CCR2	1.89176	IFIT1	1.98032
53	GBP1	9.02367	MS4A7	1.88953	PRG4	1.97931
54	HCAR3	9.01127	MX2	1.88945	BST2	1.96556
55	MMP19	8.95628	CD48	1.87189	ELAVL4	1.9616
56	TNFAIP3	8.44559	SPINK7	1.86682	CLSTN2	1.95774
57	RARRES3	8.16599	IGFL1	1.86305	BHLHE40	1.93794
58	OAS1	7.94244	SAMD9	1.85469	RNF213	1.92888
59	TRPV3	7.91645	OLR1	1.84506	DDX60	1.92631
60	NLRC5	7.83556	TIMP1	1.83962	PTPRH	1.92564
61	APOL1	7.78817	APOB	1.83878	DHX58	1.90416
62	INHBA	7.77778	PLSCR1	1.82812	IGF2	1.87869
63	XAF1	7.72118	C12orf56	1.80999	SHISA6	1.87863
64	SCG5	7.1743	BEND6	1.80616	ARG1	1.87709
65	CEACAM1	7.05259	C1QB	1.80589	KRT6A	1.87523
66	PLA2G4C	6.97243	TNFSF13B	1.80499	LDOC1	1.87442
67	IFI44	6.72954	CLEC7A	1.79727	SDC2	1.87327
68	ZPLD1	6.64969	SELL	1.79477	CHI3L1	1.86271
69	IRAK2	6.59654	IL7R	1.78607	SSC5D	1.85698
70	BPGM	6.55772	THY1	1.78432	FAM212B	1.85176
71	TAGLN3	6.5515	GDA	1.78425	AQP5	1.82811
72	IL23A	6.45744	IFIT3	1.7789	PTCHD1	1.81909
73	TNFSF10	6.44544	LTB	1.77786	HK2	1.81653
74	NAV3	6.4116	CEP55	1.77766	FETUB	1.81495
75	FA2H	6.40869	FYB	1.77189	CBLB	1.80518
76	ABCA1	6.40615	C5AR1	1.77022	MAST4	1.80388
77	SFR1	6.40135	C1QC	1.76419	DDX58	1.80246
78	LAMP3	6.36948	IGSF6	1.76306	KCTD11	1.79765
79	QPCT	6.34037	CCL19	1.762	KIAA1199	1.79737
80	MAMDC2	6.29795	LILRB2	1.76074	AKR1D1	1.79483
81	CCL20	6.29054	ARNTL2	1.74873	PIK3CB	1.79404
82	COL22A1	6.23082	APELA	1.74365	PARP14	1.79234
83	IRF1	6.12434	HRNR	1.74093	NPL	1.79225
84	NCF2	6.05205	PARP14	1.74089	ZMYM1	1.79191
85	TNFAIP2	6.052	CECR1	1.73408	SGK494	1.78518
86	CST6	6.0241	JAML	1.7282	TREX2	1.77941
87	IFI44L	5.93179	DDX60L	1.72429	SV2B	1.77911
88	CFB	5.82279	ITGB2	1.72113	GBP7	1.77869
89	C1S	5.79985	MRC1	1.72112	IL36B	1.77461
90	IFI35	5.75773	SAMD9L	1.7187	SLC30A4	1.77245
91	HLA-B	5.74582	CCL13	1.71351	CMIP	1.77111
92	ROBO4	5.7292	KIF14	1.71261	AQP3	1.77055
93	GBP4	5.63384	CDKN3	1.71185	SERPINB11	1.76601
94	TLR3	5.5927	IDO1	1.70358	FICD	1.76231
95	SLC41A2	5.58582	LAMP3	1.70176	GNS	1.76106
96	FUT2	5.57771	CD209	1.70054	CAPN6	1.75118
97	B3GNT7	5.55604	CLEC10A	1.70029	TUSC3	1.74959
98	PARP9	5.53279	XRCC4	1.69745	GRIA1	1.74585
99	CXCL2	5.49607	STAT1	1.69492	GRIK1	1.74259
100	PTGS2	5.47393	WFDC12	1.6921	CDK16	1.73287

	Gene Symbol	PolyIC vs. Vehicle Lin(FC)	Gene Symbol	Photoaged arm post laser vs. Photoaged arm Lin(FC)	Gene Symbol	Mixed (High reg.) vs. C57 (low reg.) Lin(FC)
101	C10orf10	5.46419	IFIH1	1.69197	KLK12	1.72488
102	THEMIS2	5.41064	IRF8	1.68929	RABEPK	1.72401
103	PMAIP1	5.4025	SPRR2B	1.67703	TIMD4	1.72323
104	IL13RA2	5.30581	SERPINE1	1.67047	STAT2	1.72005
105	PTPRK	5.25126	AMDHD1	1.66988	EPHA3	1.71788
106	HSD17B2	5.24459	RB1	1.66896	PAPOLB	1.71387
107	NFE2L3	5.1617	SASH3	1.6683	ZNF281	1.71308
108	IFIH1	5.15255	CR1	1.66285	PIEZO2	1.71296
109	NMNAT2	5.13203	RRM2	1.65935	FMOD	1.71208
110	HLA-C	5.1289	KIF20A	1.65832	XAF1	1.70687
111	WNT7B	5.09285	LCE3D	1.65716	TROVE2	1.70471
112	PPBP	5.07194	CD53	1.65713	SPOCK1	1.70087
113	STAT1	5.06094	ITGAM	1.65637	THBS4	1.70063
114	HERC5	4.98858	VSIG4	1.64749	CD300E	1.6995
115	OAS3	4.9713	COMP	1.64339	DSG1	1.69269
116	SLC15A3	4.95848	GZMA	1.6414	KLHL5	1.69245
117	LMO2	4.95754	KYNU	1.63493	PIANP	1.69229
118	TUBB3	4.94205	HMMR	1.62649	DUOX1	1.6922
119	TNIP1	4.93565	PLEK	1.62571	RGS4	1.6919
120	TNF	4.9337	SIGLEC1	1.62436	SP140	1.68596
121	ETV7	4.93303	C2	1.62256	INHBA	1.68114
122	AIM2	4.89951	ADAMTS12	1.61967	TMEM104	1.67932
123	SERPING1	4.89727	GPR65	1.61863	OR5D16	1.67772
124	BATF2	4.88673	SPRR1A	1.61741	MGARP	1.67647
125	DAB2	4.85796	TDO2	1.61562	TAC1	1.67556
126	PGM2L1	4.85293	SGO2	1.61184	POC1B	1.67475
127	DDX60	4.84879	FOSL1	1.61113	DLST	1.67451
128	MFI2	4.83083	DLGAP5	1.60707	COQ10B	1.67075
129	TDRD7	4.76836	POLR3G	1.60642	CCNT2	1.67033
130	TIMP2	4.76656	TLR2	1.60443	HAVCR1	1.669
131	SAMHD1	4.75406	PI3	1.60426	PARP9	1.66676
132	WFDC2	4.72625	AADAC	1.60264	CEP72	1.66659
133	HLA-A	4.71214	TNC	1.6025	MAP6D1	1.66579
134	SOCS1	4.70215	KNL1	1.59433	GRK6	1.66497
135	FAM180A	4.67439	FAP	1.59318	GBP4	1.66245
136	NMI	4.62087	MPEG1	1.59127	GLT8D1	1.66098
137	MAP3K7CL	4.61393	FCER1G	1.5889	NEO1	1.66037
138	TMEM27	4.60292	HAL	1.58604	SMAP2	1.65587
139	PARP14	4.5873	NETO2	1.58526	NXPH4	1.65479
140	ZC3HAV1	4.56106	FCGR2A	1.58423	ZNF385B	1.65378
141	GBP5	4.55759	CCR1	1.58129	KLK13	1.65009
142	PLD5	4.55376	MS4A4A	1.57821	MAPRE1	1.64744
143	FN1	4.53974	CHN1	1.57619	MAN1A1	1.64693
144	EHD1	4.53437	CLEC4A	1.57509	HERC6	1.64575
145	PSMB9	4.52926	CCR5	1.57473	LINGO2	1.64476
146	ZNF365	4.52798	JCHAIN	1.57365	ZBTB40	1.64421
147	TUBA1A	4.51095	DHRS9	1.57347	PARVG	1.6435
148	MME	4.45629	COL6A3	1.57124	SERPINA9	1.64274
149	MT1G	4.44979	ARHGAP9	1.56953	RORB	1.6413
150	DDX60L	4.44467	PMCH	1.56945	SRGAP3	1.6398
151	SEMA3C	4.43991	IL36G	1.56563	ONECUT2	1.63875
152	TRIM21	4.39953	SLC44A5	1.56485	CNFN	1.63592
153	SERPINB1	4.38529	TFEC	1.56381	ALOX12B	1.63588
154	PLAUR	4.37331	SRPX	1.56076	HELZ2	1.63472
155	SPHK1	4.36634	DCANP1	1.56067	DTX3L	1.63121
156	ADAM19	4.35733	ITGAL	1.55859	SPSB2	1.62958
157	CEACAM6	4.33672	TCHH	1.55708	GJD2	1.62886
158	DPP4	4.33531	IRF9	1.55605	FBXO30	1.62847
159	DEFB103B	4.32461	DTX3L	1.55485	IFI35	1.62811
160	IFNB1	4.3221	HPSE	1.55429	RIM6-TRIM	1.62776
161	C20orf112	4.30838	GZMB	1.5536	SPTBN4	1.62618
162	LAMA2	4.28691	COTL1	1.55348	C10orf118	1.62578
163	OAS2	4.23652	GREM1	1.55273	LYST	1.62548
164	IL36G	4.19885	MNDA	1.55272	ZBTB24	1.62367
165	TFPI	4.1916	CERKL	1.55182	PTX4	1.62206
166	RNASE7	4.18987	CD3D	1.54796	PLAC8	1.62073
167	ISG15	4.18664	MARCH1	1.54684	IRF9	1.62049
168	DEFB4A	4.18195	CORIN	1.54404	TRIM5	1.61752
169	TM4SF19	4.17617	HERC5	1.54329	DBC1	1.61409
170	GJB4	4.16099	BCAT1	1.54195	SPON1	1.60993
171	PLSCR1	4.15245	CD68	1.54037	TRIM10	1.60792
172	C1orf74	4.09941	KIAA0101	1.54022	CXCL9	1.60751
173	PRDM8	4.07307	AIF1	1.53981	BNIP3L	1.60675
174	DTX3L	4.03377	GTSE1	1.53912	GRIA2	1.60381
175	TBX3	4.03025	LCE3E	1.5363	MMP9	1.60372
176	SLC2A12	4.01971	CENPE	1.53581	PRODH	1.60346
177	ITGA5	4.011	KCNJ15	1.53335	C15orf57	1.60318
178	GBP3	3.99692	NMI	1.53301	HBEGF	1.60247
179	GMPR	3.98835	SERPINA1	1.53211	SEC14L1	1.60182
180	SOX9	3.97898	IL33	1.53117	KIF22	1.60014
181	ACE2	3.95463	IFITM1	1.5279	CD300LF	1.59914
182	L1CAM	3.94496	MKI67	1.52578	LGALS3BP	1.5991
183	EPHX4	3.93269	ST8SIA4	1.52279	SMARCA1	1.5978
184	BTN2A2	3.92137	GABBR1	1.52193	ANAPC2	1.59711
185	WFDC3	3.91183	CTSC	1.52059	YIPF6	1.59707
186	HERC6	3.8829	TRAT1	1.52034	C12orf66	1.59673
187	ANO4	3.872	RTP4	1.52029	HTR2A	1.59612
188	IL7R	3.85129	AOAH	1.51918	DDX56	1.59596
189	VNN1	3.8392	GBP1	1.51786	FCGR1A	1.59374
190	ZNFX1	3.81918	SLC7A11	1.51697	HIC2	1.59226
191	MGLL	3.81705	GPR183	1.51675	NKX6-2	1.59151
192	SERPINB9	3.81179	RAD51AP1	1.51595	CMPK2	1.58949
193	CARD16	3.80683	ADAP2	1.51461	PCDH11Y	1.58557
194	HELZ2	3.80293	ASPM	1.51097	PCDH11X	1.58557
195	PML	3.80062	SP110	1.51063	COL24A1	1.58548
196	HDAC9	3.77386	TPX2	1.51044	MPP4	1.58486
197	FMN1	3.76575	PIK3AP1	1.50876	GNAO1	1.58373
198	ABCG1	3.76549	CFB	1.50789	MPP3	1.58364
199	GLRX	3.67878	LRMP	1.50763	RBM3	1.58342
200	DNAJB5	3.66839	RAB39A	1.50522	MTX1	1.581

S2

A

	Gene Symbol	*Rnasel-/- vs. WT Abundance Ratio*
1	Atg4b	1000
2	Mrps35	1000
3	Celf1	1000
4	Tktl2	1000
5	Krt28	1000
6	Esp31	1000
7	Sprr2h	1000
8	Cox17	1000
9	Tp53bp1	1000
10	Pdcd4	1000
11	Fam25c	1000
12	Dhcr24	1000
13	Nek7	1000
14	Krt26	18.011
15	Ttc1	14.73
16	Krt4	3.316
17	Krt84	3.071
18	Krt36	2.884
19	Ccdc9	2.857
20	Myt1	2.547
21	Snrpc	2.384
22	Mt2	2.348
23	Rpa1	2.222
24	Sprr2f	2.217
25	Abraxas2	2.092
26	Plscr3	2.049
27	Ensa	2.047
28	Sprr1b	2.043
29	Mt1	1.991
30	Galk2	1.931
31	Ly6a	1.924
32	Flg	1.884
33	Vkorc1	1.848
34	Myh10	1.819
35	Crct1	1.764
36	Gsdma	1.759
37	Ech1	1.728
38	Htra1	1.714
39	Cryab	1.709
40	Sprr1a	1.709
41	Slc25a22	1.701
42	Eif4ebp2	1.682
43	Gja1	1.677
44	Rae1	1.651
45	Hexb	1.63
46	Aldoart1	1.604
47	Ankrd22	1.595
48	Itm2b	1.579
49	Snap47	1.578
50	Degs1	1.574

	Gene Symbol	*Rnasel-/- vs. WT Abundance Ratio*
51	Dsg1b	1.568
52	Sdc1	1.565
53	Cnfn	1.563
54	Mob2	1.556
55	Baiap2	1.545
56	Hmcn1	0.643
57	Epn1	0.633
58	Epb41l3	0.628
59	Cers6	0.621
60	Snrpb2	0.619
61	Tm9sf4	0.617
62	Txlna	0.615
63	Apex2	0.611
64	Slc7a5	0.6
65	Ptma	0.596
66	Itga2	0.596
67	Sympk	0.582
68	Mepce	0.569
69	Rrp9	0.568
70	Bop1	0.561
71	Amot	0.555
72	Hmga2	0.551
73	Acot2	0.532
74	Ggt7	0.531
75	Ppp4r3a	0.524
76	Fam3c	0.517
77	Ecsit	0.508
78	Krt8	0.504
79	Ifi202	0.504
80	Vkorc1l1	0.488
81	Iap	0.47
82	Crocc	0.461
83	Ptgs2	0.452
84	Ano6	0.452
85	Mpp7	0.449
86	Snrpe	0.422
87	Nalcn	0.421
88	Fam129a	0.4
89	Slc1a4	0.382
90	Hars2	0.351
91	Slc35f6	0.35
92	Ndufs6	0.344
93	Akap10	0.325
94	Ccdc127	0.301
95	Tmigd3	0.28
96	Isg15	0.279
97	Cpt1b	0.279
98	U2af1	0.261
99	Cd151	0.223
100	Cep162	0.152

B

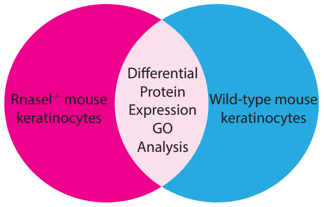

GO terms enriched in proteomics (Rnasel$^{-/-}$ vs. WT keratinocytes)

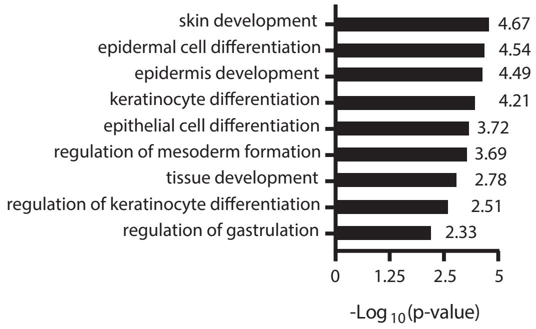

S3

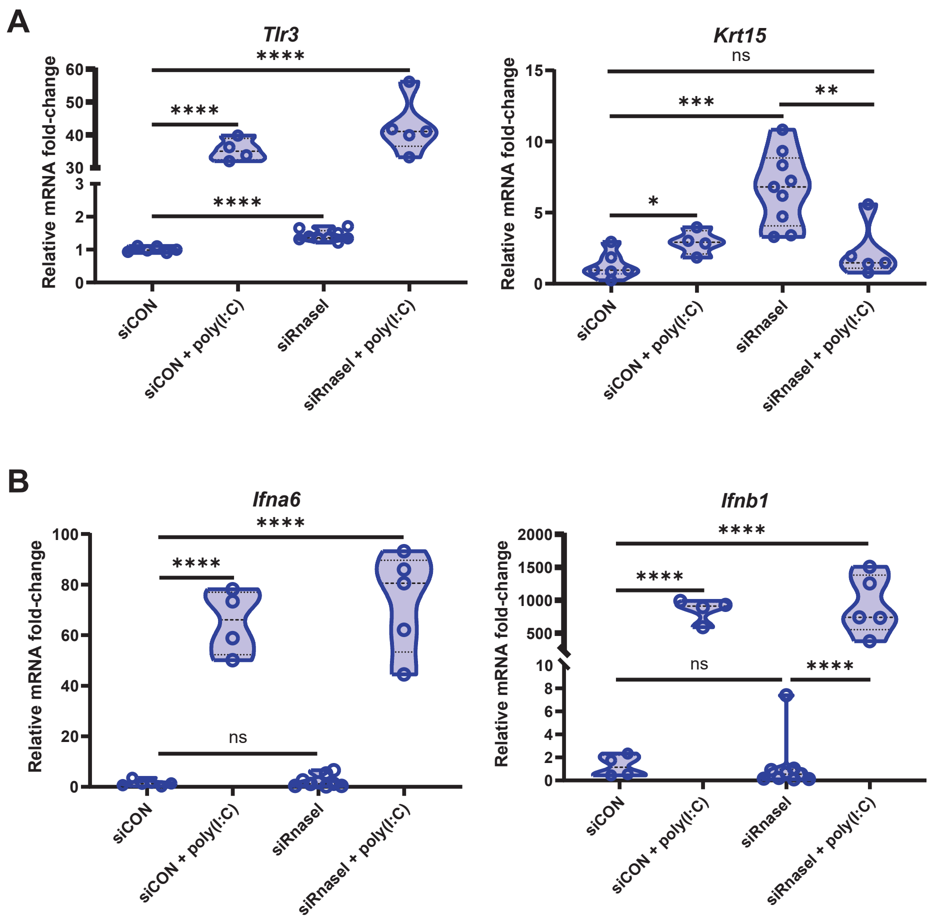
A
Tlr3
Relative mRNA fold-change
60
50
40
30
3
2
1
0
****
****
****
siCON
siCON + poly(I:C)
siRnasel
siRnasel + poly(I:C)
Krt15
Relative mRNA fold-change
15
10
5
0
ns
***
**
*
B
Ifna6
Relative mRNA fold-change
100
80
60
40
20
0
****
****
ns
Ifnb1
Relative mRNA fold-change
2000
1500
1000
500
10
8
6
4
2
0
****
****
ns
****

S4

A

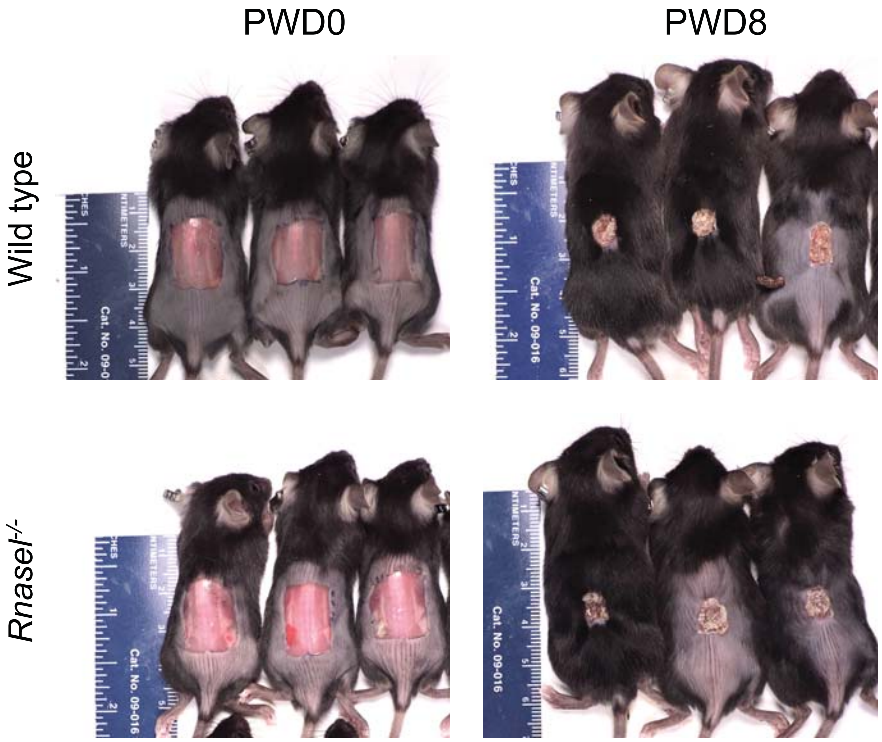

S5

A

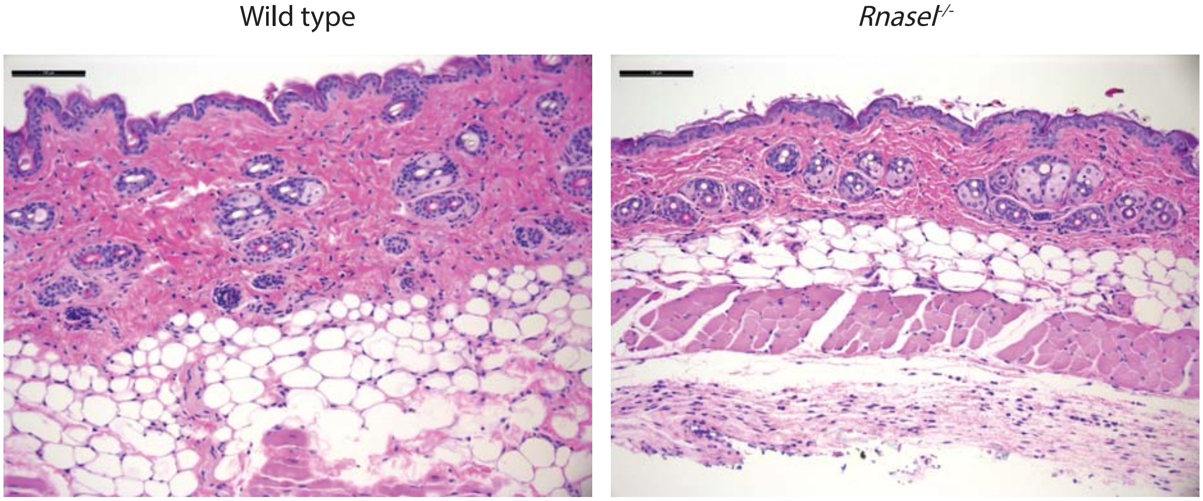

Wild type
Rnasel-/-

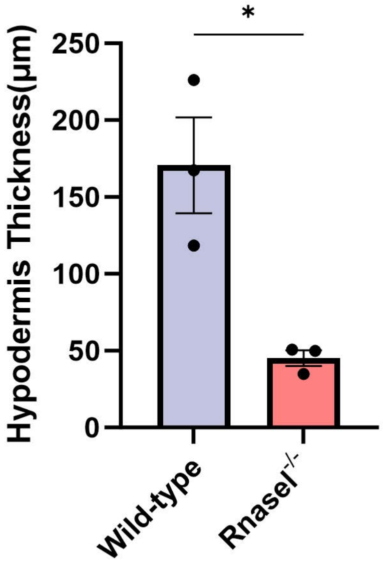

Hypodermis Thickness(μm)
250
200
150
100
50
0
*
Wild-type
Rnasel-/-

S6

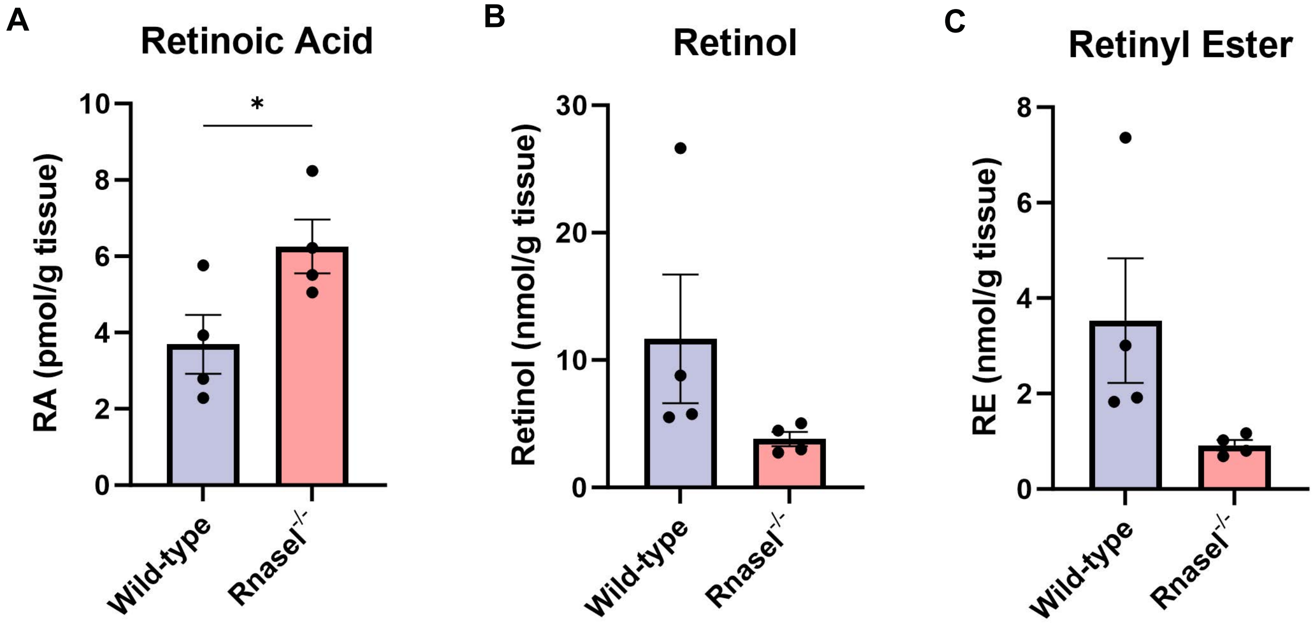
A
Retinoic Acid
RA (pmol/g tissue)
10
8
6
4
2
0
*
Wild-type
Rnasel-/-
B
Retinol
Retinol (nmol/g tissue)
30
20
10
0
Wild-type
Rnasel-/-
C
Retinyl Ester
RE (nmol/g tissue)
8
6
4
2
0
Wild-type
Rnasel-/-

S7

A

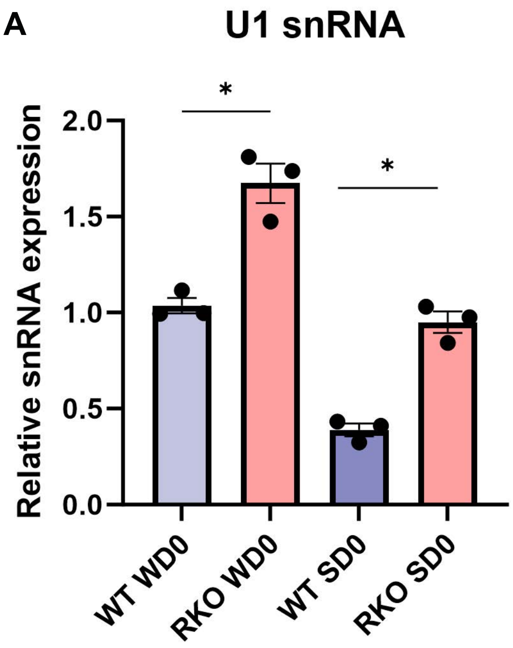
U1 snRNA
Relative snRNA expression
2.0
1.5
1.0
0.5
0.0
*
*
WT WD0
RKO WD0
WT SD0
RKO SD0

S8 **A**

	Gene Symbol	***Rnasel-/- vs. WT Lin(FC)***
1	Akr1c14	10.0021
2	Sfrp4	8.13652
3	Retnla	7.71007
4	Pi16	7.60366
5	Ankhd1	5.62126
6	Lbp	5.50304
7	Cyp2g1	4.98483
8	Ipw	4.96414
9	Akr1c18	4.93945
10	Myl1	4.62749
11	Crmp1	4.57394
12	Sod3	4.29343
13	Ear12	4.13806
14	Mup19	4.01317
15	Mup8	3.99241
16	Npy1r	3.98656
17	Mup1	3.95508
18	Gzmc	3.94036
19	Mup13	3.87464
20	Mup12	3.86668
21	Mgl2	3.8049
22	Plppr4	3.77747
23	Krt24	3.77619
24	Mup7	3.74386
25	Folr2	3.72536
26	Cilp	3.65451
27	Clec3b	3.63942
28	Pgr	3.61046
29	Cyp2f2	3.57958
30	AA467197	3.55591
31	Lrrn1	3.54855
32	C3	3.50004
33	F13a1	3.476
34	Mup2	3.41595
35	Gm2083	3.41595
36	Ttr	3.37843
37	Mup2	3.37434
38	St3gal1	3.36248
39	Mmp12	3.34372
40	Gadl1	3.32136
41	Gas6	3.30276
42	Fcrls	3.28463
43	Ccl9	3.28362
44	Cyp17a1	3.26735
45	Fos	3.21163
46	Cd55	3.20336
47	Tnxb	3.16909
48	Cd209d	3.08186
49	Mgp	3.03748
50	Myh1	3.03672
51	Prss23	3.02594
52	Mrc1	3.02023
53	C4a	3.01645
54	Dusp1	3.01495
55	Selp	3.01444
56	Thbs3	3.01182
57	Rtn1	3.01106
58	C4b	2.96362
59	Cygb	2.94847
60	Ccl8	2.93798
61	Ace	2.93708
62	Gp1ba	2.91
63	Cfh	2.89308
64	Snap25	2.88671
65	Nxpe5	2.8738
66	Actg2	2.86858
67	Cpxm2	2.86795
68	Atp6v1b1	2.84958
69	Kmo	2.83568
70	Pltp	2.83488
71	Serping1	2.82026
72	Nr1d1	2.81051
73	Etv1	2.77704
74	Ackr3	2.77611
75	Ina	2.75538
76	Fmod	2.74515
77	Dok2	2.73937
78	Pla1a	2.73317
79	Ar	2.7276
80	Igfbp6	2.72619
81	Scn3b	2.71967
82	Il31ra	2.70833
83	Prlr	2.69999
84	Fcgrt	2.69833
85	Itgb2	2.67685
86	4930449A1	2.65037
87	Dbp	2.64384
88	Gpx3	2.64334
89	Cbr2	2.61708
90	Clec10a	2.59347
91	Rgs4	2.58394
92	Eln	2.57183
93	Panx3	2.56737
94	Kcna6	2.56736
95	Nsg1	2.56083
96	Olfr1364	2.54534
97	H2-M2	2.53956
98	Ramp2	2.53895
99	Des	2.53534
100	Ntrk2	2.52882

	Gene Symbol	***Rnasel-/- vs. WT Lin(FC)***
101	Prox2	2.52719
102	Fcgbp	2.52356
103	Emilin2	2.52136
104	Chrdl1	2.51444
105	St3gal6	2.50764
106	Tcaf2	2.49804
107	Prelp	2.49111
108	Myh8	2.4817
109	Pmp22	2.48167
110	Cd226	2.47994
111	Ly6a	2.47571
112	Tmeff2	2.47119
113	Cfp	2.46085
114	Efemp1	2.45947
115	Emb	2.45312
116	Cadm3	2.45252
117	Ska3	2.44563
118	Cd209g	2.43718
119	Ccl11	2.43658
120	Kcnk5	2.43658
121	Scgb1a1	2.43338
122	Itgam	2.4328
123	Fgf7	2.43014
124	Kcnma1	2.42592
125	Lepr	2.42331
126	C1ra	2.42038
127	Gm973	2.42021
128	Gng3	2.41991
129	Krt77	2.41639
130	Ttc39a	2.4153
131	Gm13304	2.41343
132	Ccl21b	2.41343
133	Krt2	2.4133
134	Clec4a3	2.40963
135	Il33	2.40794
136	Entpd2	2.40543
137	Mmp3	2.40353
138	Ang2	2.39329
139	Scn7a	2.39315
140	Slc16a10	2.38597
141	Alx4	2.37347
142	Serpina3n	2.3718
143	Myh2	2.36606
144	Atp8b1	2.36204
145	Zfp872	2.36155
146	H2-DMb1	2.35423
147	H2-DMa	2.35318
148	Hsd3b6	2.3511
149	Adam23	2.34109
150	Cnn1	2.33806
151	Galnt12	2.3348
152	Mrgprb1	2.32793
153	Pnpla5	2.32761
154	Crip1	2.31766
155	H2-Aa	2.30961
156	Serpinb6e	2.30475
157	Uchl1	2.30241
158	Naaa	2.30232
159	Ly6c1	2.30112
160	Car11	2.29939
161	Ccl6	2.29005
162	Pirb	2.28906
163	Fabp7	2.28174
164	Mustn1	2.28131
165	Ear10	2.27892
166	Gm16485	2.2774
167	Snx10	2.27194
168	Ccl21a	2.2703
169	Lrrn4cl	2.26743
170	Lgi2	2.26605
171	Fbln1	2.258
172	Gm13304	2.25334
173	Gm13304	2.25334
174	Ccl21c	2.25334
175	C5ar1	2.25265
176	Clec5a	2.24857
177	Steap3	2.24828
178	Gm13304	2.24443
179	Gm1987	2.24443
180	Gm10591	2.24443
181	Adh1	2.23362
182	Gm15097	2.22132
183	Sfrp2	2.21734
184	Abca6	2.21281
185	Dkk2	2.20894
186	Mup3	2.19928
187	Stmn4	2.19684
188	Timp3	2.19428
189	Fa2h	2.19343
190	Trim47	2.19082
191	Mapk10	2.18982
192	Itgax	2.18494
193	Klrb1c	2.18172
194	Gm9972	2.18122
195	Nov	2.18047
196	Ptgs1	2.17818
197	Mcpt4	2.17238
198	Serpina3j	2.17137
199	Plekha2	2.16862
200	Ralgps1	2.16806

S9

A

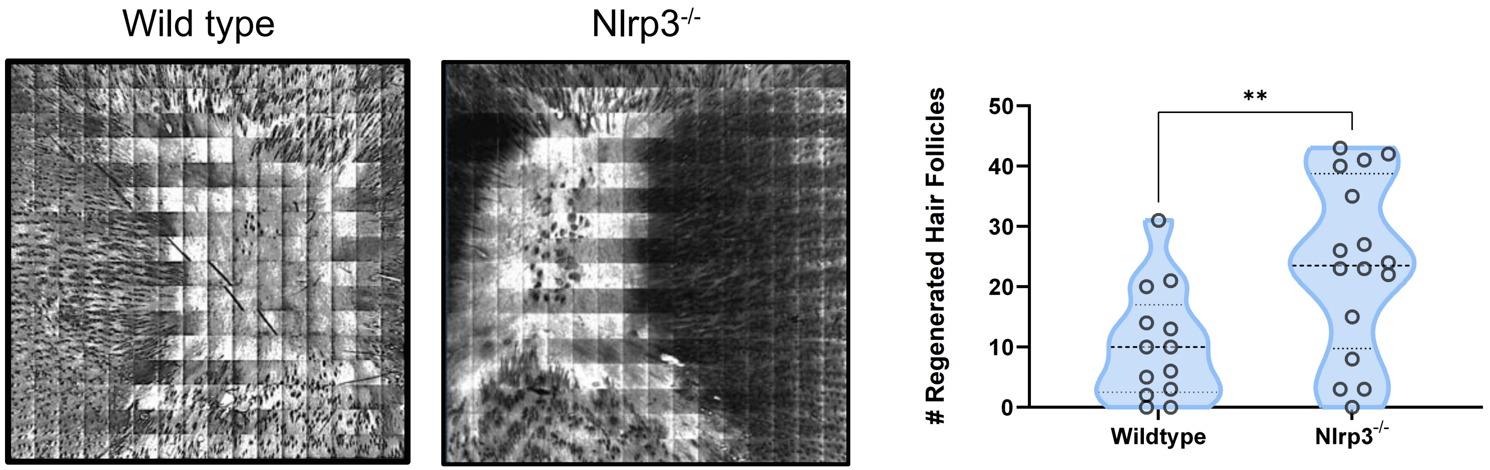

Wild type
Nlrp3-/-
Regenerated Hair Follicles
50
40
30
20
10
0
**
Wildtype
Nlrp3-/-

B

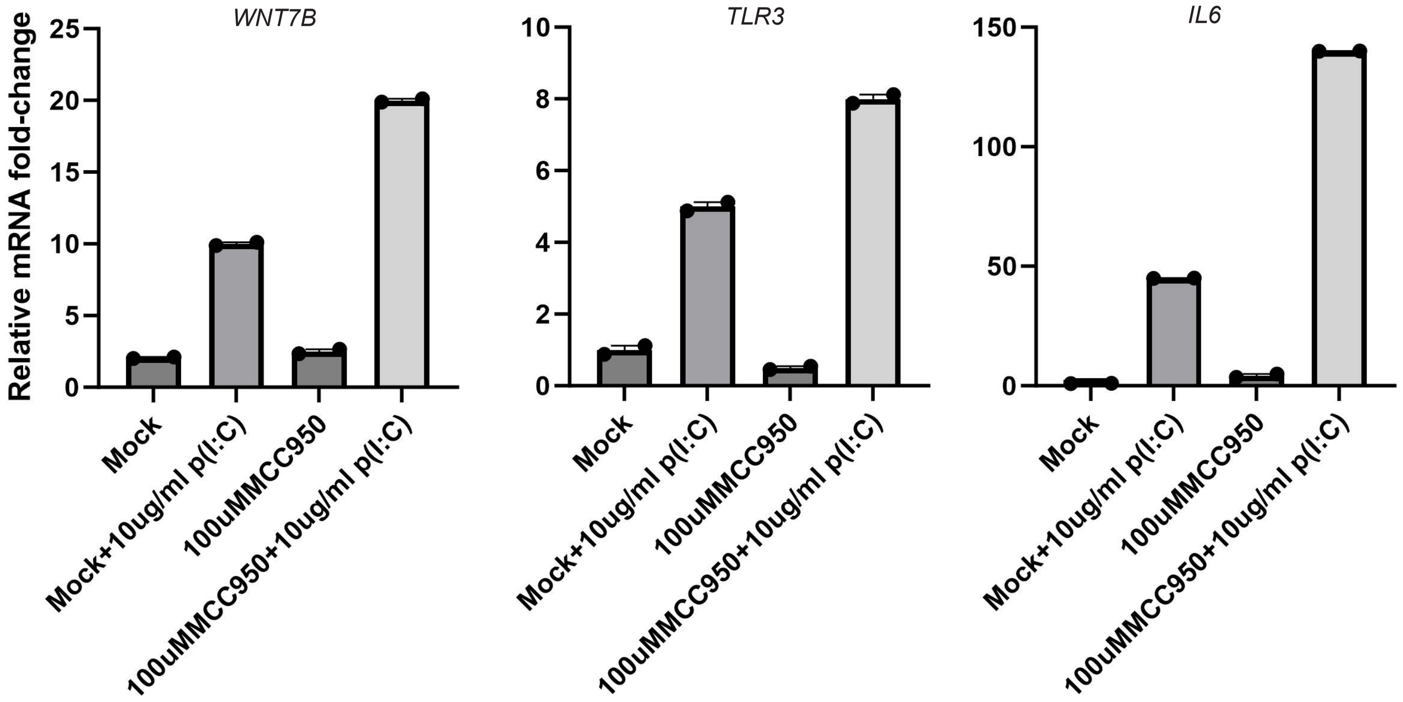

Relative mRNA fold-change
WNT7B
25
20
15
10
5
0
TLR3
10
8
6
4
2
0
IL6
150
100
50
0
Mock
Mock+10ug/ml p(I:C)
100uMMCC950
100uMMCC950+10ug/ml p(I:C)

S10

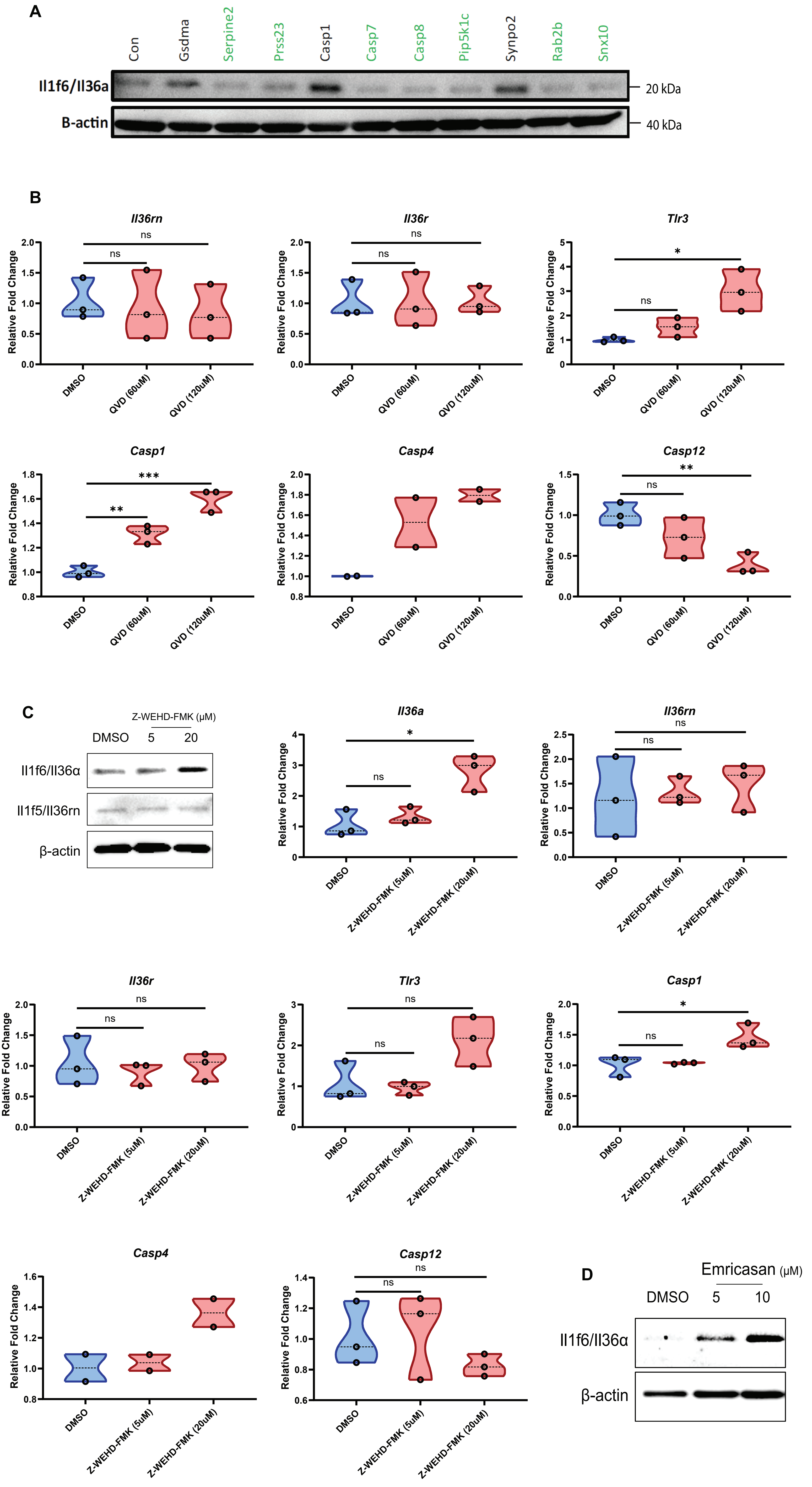
A
Con
Gsdma
Serpine2
Prss23
Casp1
Casp7
Casp8
Pip5k1c
Synpo2
Rab2b
Snx10
Il1f6/Il36a
B-actin
20 kDa
40 kDa
B
Il36rn
Il36r
Tlr3
Casp1
Casp4
Casp12
Relative Fold Change
DMSO
QVD (60uM)
QVD (120uM)
ns
*
**
***
C
Z-WEHD-FMK (μM)
DMSO
5
20
Il1f6/Il36α
Il1f5/Il36rn
β-actin
Il36a
Il36rn
Il36r
Tlr3
Casp1
Casp4
Casp12
Z-WEHD-FMK (5uM)
Z-WEHD-FMK (20uM)
D
Emricasan (μM)
DMSO
5
10
Il1f6/Il36α
β-actin

S11

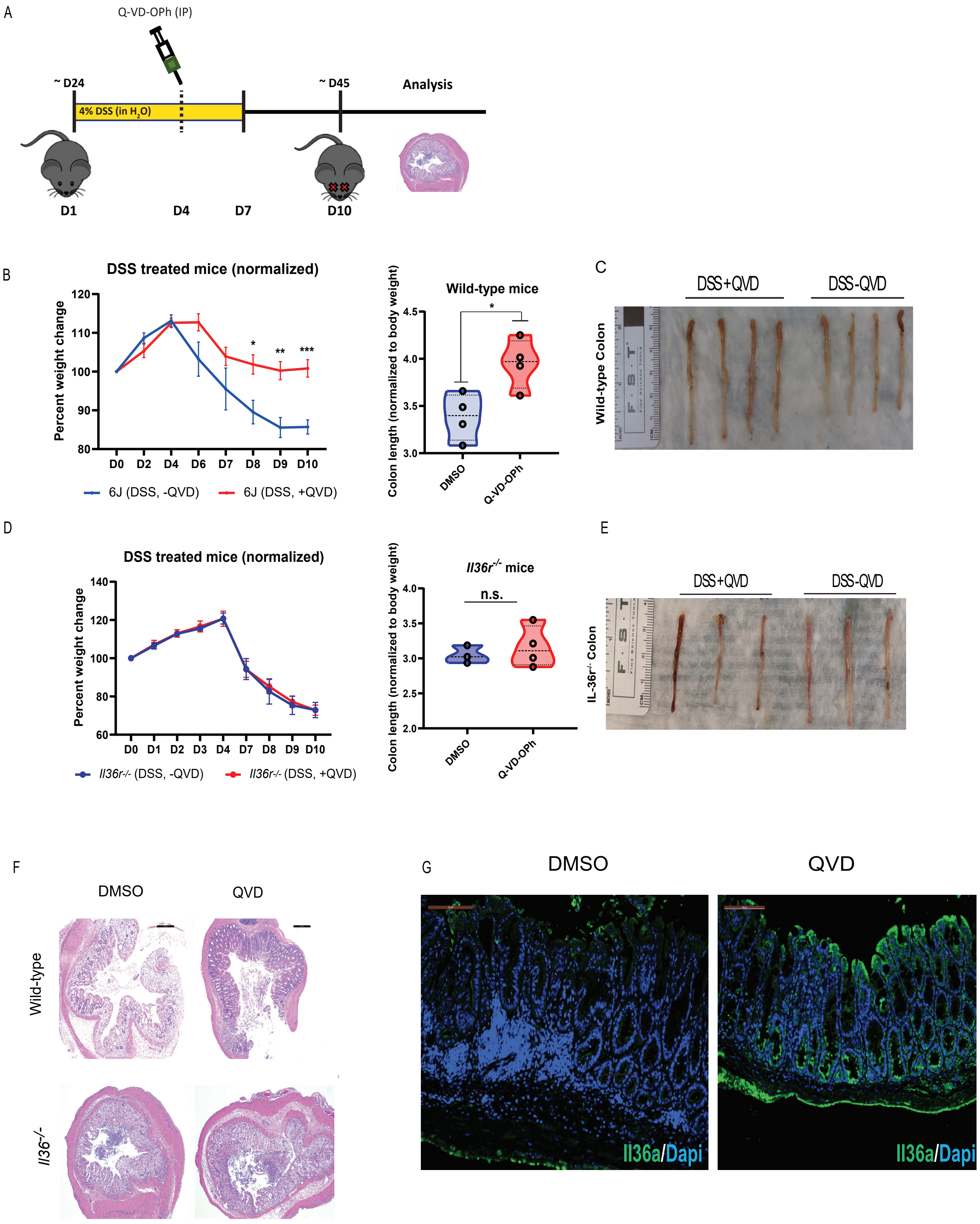
A
Q-VD-OPh (IP)
~ D24
4% DSS (in H2O)
~ D45
Analysis
D1
D4
D7
D10
B
DSS treated mice (normalized)
Percent weight change
120
110
100
90
80
D0 D2 D4 D6 D7 D8 D9 D10
*
**
***
6J (DSS, -QVD)
6J (DSS, +QVD)
Wild-type mice
Colon length (normalized to body weight)
4.5
4.0
3.5
3.0
*
DMSO
Q-VD-OPh
C
DSS+QVD
DSS-QVD
Wild-type Colon
D
DSS treated mice (normalized)
Percent weight change
140
120
100
80
60
D0 D1 D2 D3 D4 D7 D8 D9 D10
Il36r-/- (DSS, -QVD)
Il36r-/- (DSS, +QVD)
Il36r-/- mice
n.s.
Colon length (normalized to body weight)
4.0
3.5
3.0
2.5
2.0
DMSO
Q-VD-OPh
E
DSS+QVD
DSS-QVD
IL-36r-/- Colon
F
DMSO
QVD
Wild-type
Il36-/-
G
DMSO
QVD
Il36a/Dapi
Il36a/Dapi

S12

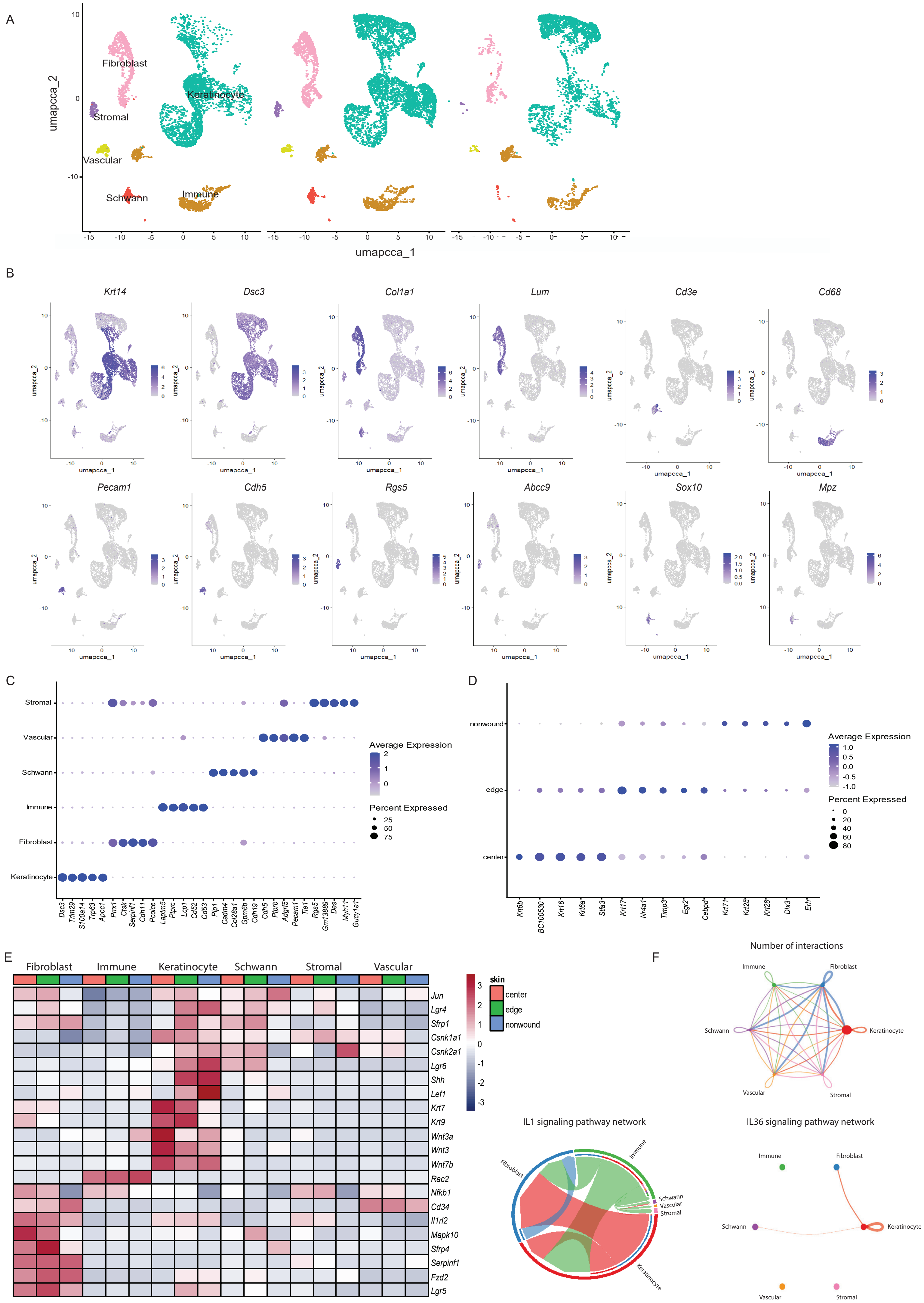

A
Center
Edge
Nonwound
Fibroblast
Keratinocyte
Stromal
Vascular
Schwann
Immune
umapcca_2
umapcca_1
B
Krt14
Dsc3
Col1a1
Lum
Cd3e
Cd68
Pecam1
Cdh5
Rgs5
Abcc9
Sox10
Mpz
C
Stromal
Vascular
Schwann
Immune
Fibroblast
Keratinocyte
Average Expression
Percent Expressed
Dsc3 Trim29 S100a14 Trp63 Apoc1 Prrx1 Ctsk Serpinf1 Cdh11 Pcolce Laptm5 Ptprc Lcp1 Cd52 Cd53 Plp1 Cadm4 Col28a1 Gpm6b Cdh19 Cdh5 Ptprb Adgrf5 Pecam1 Tie1 Rgs5 Gm13889 Des Myh11 Gucy1a1
D
nonwound
edge
center
Average Expression
Percent Expressed
Krt6b BC100530 Krt16 Krt6a Sfn3 Krt17 Nr4a1 Timp3 Egr2 Cebpd Krt71 Krt25 Krt28 Dlx3 Ezh
E
Fibroblast Immune Keratinocyte Schwann Stromal Vascular
skin
center
edge
nonwound
Jun Lgr4 Sfrp1 Csnk1a1 Csnk2a1 Lgr6 Shh Lef1 Krt7 Krt9 Wnt3a Wnt3 Wnt7b Rac2 Nfkb1 Cd34 Il1rl2 Mapk10 Sfrp4 Serpinf1 Fzd2 Lgr5
F
Number of interactions
Immune
Fibroblast
Schwann
Keratinocyte
Vascular
Stromal
IL1 signaling pathway network
IL36 signaling pathway network

S13

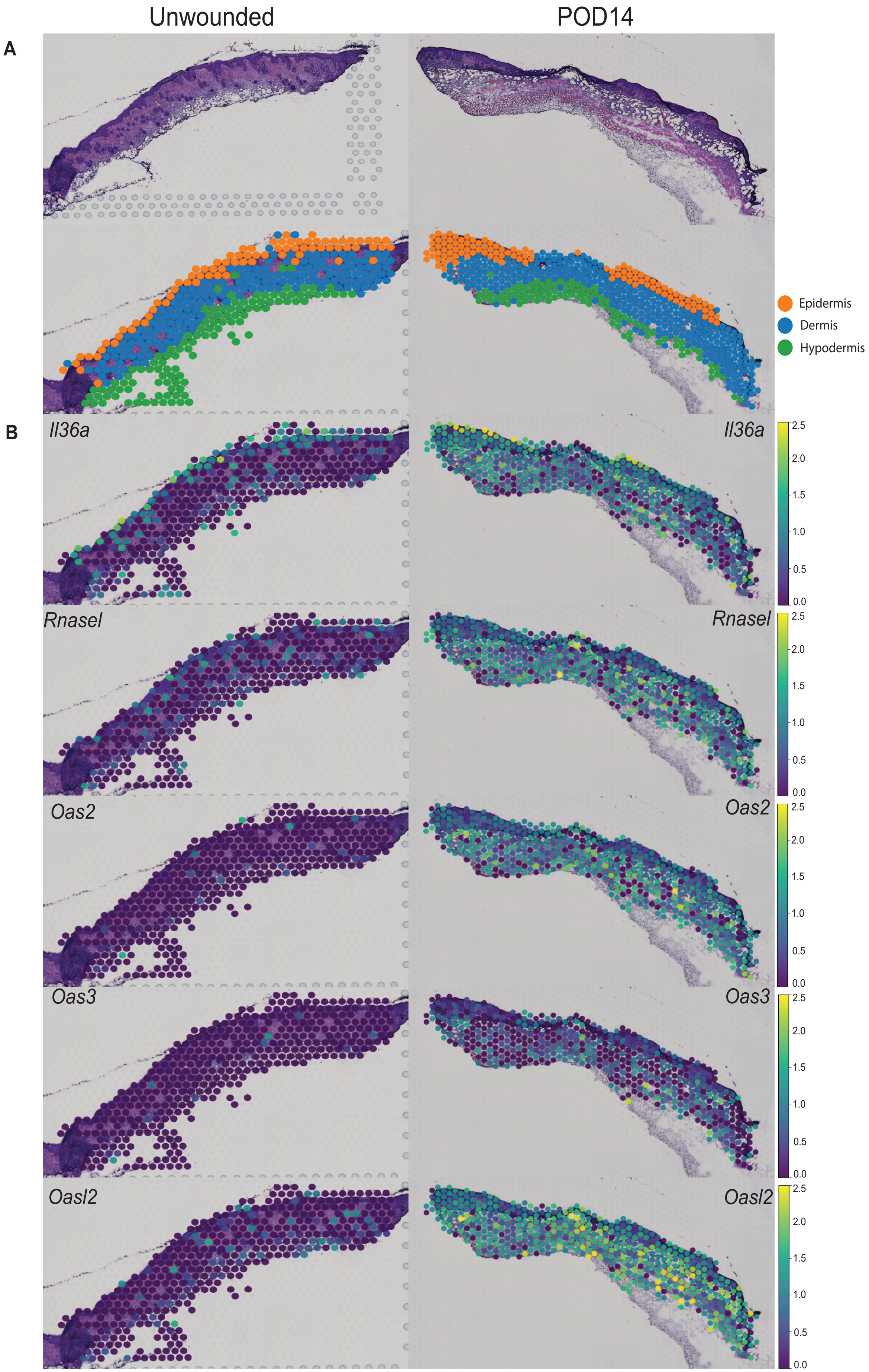

Unwounded
POD14
A
Epidermis
Dermis
Hypodermis
B
Il36a
Rnasel
Oas2
Oas3
Oasl2
2.5
2.0
1.5
1.0
0.5
0.0

S14

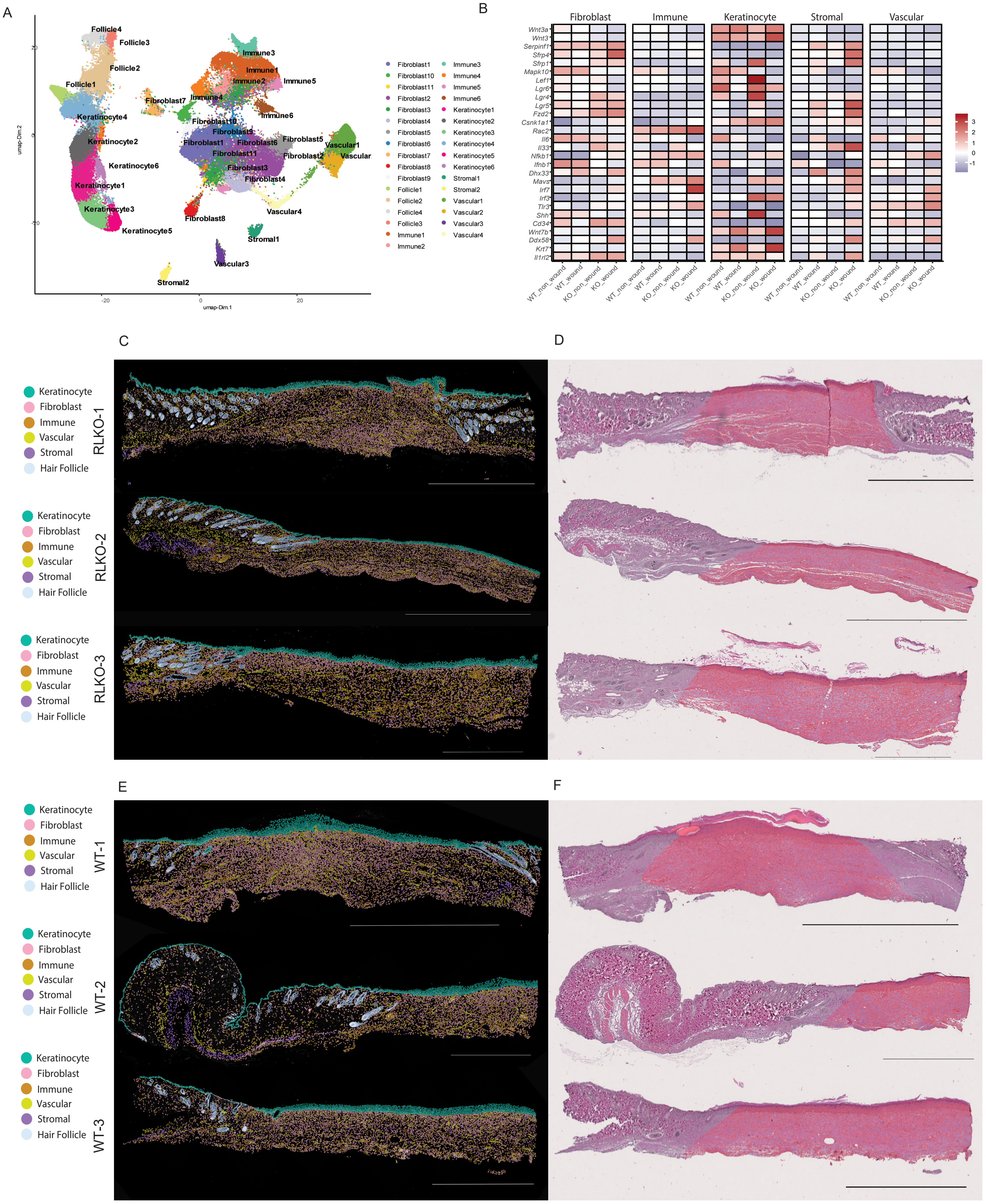

A
B
C
D
E
F
umap-Dim.1
umap-Dim.2
Follicle1
Follicle2
Follicle3
Follicle4
Keratinocyte1
Keratinocyte2
Keratinocyte3
Keratinocyte4
Keratinocyte5
Keratinocyte6
Fibroblast1
Fibroblast2
Fibroblast3
Fibroblast4
Fibroblast5
Fibroblast6
Fibroblast7
Fibroblast8
Fibroblast9
Fibroblast10
Fibroblast11
Immune1
Immune2
Immune3
Immune4
Immune5
Immune6
Stromal1
Stromal2
Vascular1
Vascular2
Vascular3
Vascular4
Fibroblast
Immune
Keratinocyte
Stromal
Vascular
Wnt3a
Wnt3
Serpinf1
Sfrp4
Sfrp1
Mapk10
Lef1
Lgr6
Lgr4
Lgr5
Fzd2
Csnk1a1
Rac2
Il6
Il33
Nfkb1
Ifnb1
Dhx33
Mavs
Irf7
Irf3
Tlr3
Shh
Cd34
Wnt7b
Ddx58
Krt7
Il1rl2
WT_non_wound
WT_wound
KO_non_wound
KO_wound
3
2
1
0
-1
Keratinocyte
Fibroblast
Immune
Vascular
Stromal
Hair Follicle
RLKO-1
RLKO-2
RLKO-3
WT-1
WT-2
WT-3

Extended data Figure S1. Top 200 genes in compared arrays

a. The top 200 genes in microarrays as described in Figure 1a. The in vitro and in vivo human microarrays contain a total of 49395 annotated transcripts each and the in vivo murine microarray contains 53145 transcripts. There is a predominance of OAS family members and OAS associated transcripts shared in each data set. These 14 genes are highlighted.

Extended data Figure S2. Proteomics analysis of WT versus Rnasel-/- keratinocytes

a. The top 100 proteins elevated in Rnasel-/- keratinocytes compared to wild type keratinocytes.

b. The genes in (a) were analyzed using gene ontology and show significant upregulation of biological processes for developmental and morphogenesis pathways.

Extended data Figure S3. RNase L loss in mouse epithelial keratinocytes induces morphogenesis markers without affecting interferon levels.

a-b. siRNA-mediated RNase L loss in mouse epithelial keratinocytes induced multiple morphogenesis markers (a), while not affecting levels of interferons (b), with and without poly (I:C) treatment (10μg/ml, 48 hrs) (n=3, 1-way ANOVA, $p<0.05$).

Extended data Figure S4. Rnasel-/- mice have normal wound closure kinetics.

a. Grossly, *Rnasel-/-* mice display normal wound closure speed. Images from wound days 0 and 8 (n=10 vs 4).

Extended data Figure S5. Rnasel-/- mice have a thinner hypodermal layer

a. Hematoxylin and eosin staining of unwounded tissue from wild type and Rnasel-/- mice show that Rnasel-/- mice have a thinner hypodermis compared to wild-type mice (n=3, 2-tailed unpaired t test, $p<0.05$). Black scale bar = 100 μm.

Extended data Figure S6. Rnasel-/- mice have elevated levels of Retinoic Acid in skin

a. Quantitation and analysis of retinoic acid (RA) levels demonstrate more RA is present in Rnasel-/- compared to wild-type mice in unwounded skin (n=4, 2-tailed unpaired t test, $p<0.05$). Measurements were acquired via LC-MS. **b.** Quantitation and analysis of retinol (ROL) levels in unwounded skin of wild type and Rnasel-/- mice does not show a similar change (n=4, 2-tailed unpaired t test, p=n.s.). **c.** Quantitation and analysis of retinyl ester (RE) levels in unwounded skin of wild-type and Rnasel-/- mice is not significant (n=4, 2-tailed unpaired t test, p<n.s.).

Extended data Figure S7. Rnasel-/- mice have elevated levels of endogenous U1 snRNA

a. U1 snRNA was measured in both unwounded and healed wounds of wild type and Rnasel-/- mice via qRT-PCR using custom TaqMan probes. Compared to wild type mice, Rnasel-/- mice express significantly higher U1 snRNA than wild-type mice (n=3, 2-way ANOVA, $p<0.05$). U6 snRNA was used as a housekeeping control.

Extended data Figure S8. Top 200 genes in Rnasel-/- versus wild type mice

a. The top 200 genes in *Rnasel-/-* mice during re-epithelialization (~10 days post-wounding) as described in Figure 3A.

Extended data Figure S9. Nlrp3-/- mice have enhanced WIHN

a. Nlrp3-/- mice exhibit increased wound induced hair neogenesis (WIHN) when compared to wild type age-matched control mice. (CSLM images; n= 13 versus 16 each, p=0.0093).

b. Chemical inhibition of NLRP3 by MCC950 (100uM) in human keratinocytes treated with poly (I:C) increased expression of morphogenesis markers(n=2).

Extended data Figure S10. Caspase inhibition enhances Il36α levels in mouse epithelial keratinocytes

a. siRNA screen in mouse keratinocytes identifying loss of caspase-1 induces IL-36α protein expression **b.** Il36rn and Il36r expression was not affected by caspase inhibition in mouse epithelial keratinocytes treated with QV-D-OPh for 48 hrs. Tlr3 and inflammatory caspases 1 and 4 were elevated after QV-D-OPh treatment (n=3, 1-way ANOVA, $p<0.05$; n=2). **c.** Il-36α protein levels, but not the receptor antagonist Il-36rn, were increased in whole-cell lysates of mouse epithelial keratinocytes treated with Group 1 caspase inhibitor Z-WEHD-FMK. Il36rn and Il36r expression was not affected by caspase inhibition in mouse epithelial keratinocytes treated with Z-WEHD-FMK for 48 hrs. Tlr3 and inflammatory caspases 1 and 4 are elevated after Z-WEHD-FMK (n=3, 1-way ANOVA, $p<0.05$; n=2). **d.** Il-36α protein levels were increased in whole-cell lysates of mouse epithelial keratinocytes treated with pan caspase inhibitor Emericasan. Results are representative of three independent experiments.

Extended data Figure S11. Caspase inhibition promotes gut regeneration in DSS-treated mice

a. Schematic of Q-VD-OPh intraperitoneal injection (1.33mM) in 4% DSS treated C57BL/J6 mice. **b**. Q-VD-OPh treatment is able to rescue weight loss in a DSS-induced colitis model. After 6 days of DSS treatment, mice begin exhibiting drastic changes in QVD versus vehicle treated mice (n=4, 2-tailed unpaired t test, $p<0.05$). **c**. Q-VD-OPh-treated mice have longer colon lengths compared to vehicle after DSS-induced damage (n=4, 2-tailed unpaired t test, p=0.0203).

Colon lengths were normalized to their starting body weight **d.** Q-VD-OPh does not rescue weight loss in *Il36r-/-* mice (n=3 vs 4). **e.** Q-VDOPh does not rescue gut shortening after DSS treatment in in *Il36r-/-* mice (n=3 vs 4). **f.** Histology of colon sections in (c) and (e) showing gross improvement of tissue after pan-caspase inhibition in wild-type, but not *Il36r-/-* mice. **g.** Q-VD-OPh induces IL36α expression (green) in the colon as it does in skin.

Extended data Figure S12. Single cell RNA-seq analysis of mouse wounded and non wounded skin

a.Umap-cca plot shows gene expression profiles of SD0 wound center, edge and nonwound peripheral skin. A total of 6 clusters were grouped together, with different clusters shown in different colors. **b**. Marker gene expression within different celltypes. **c**. Top 5 differently expressed genes within each cell type, size of the dot represents the percentage of cells expressing the gene, while the color intensity indicates the gene's average expression level. **d**. Top 5 differently expressed genes within each sample condition. **e**. Heatmap of morphogenic, IL36 pathway, immune and WNT genes (rows represent independent samples, color scale based on Z-score distribution) **f**. Celltype interactions for IL1 and IL36 pathways as detected by CellChat algorithm

Extended data Figure S13. Spatial transcriptomics of mouse wounded and unwounded skin

a.Histology & delineation of skin layers based on marker genes and histology for unwounded (POD0) and wounded (POD14) mouse skin. **b**. Gene expression activity for genes of interest (*Il36a, Rnasel, Oas2, Oas3, Oasl2*) that were expressed in both unwounded and wounded sample conditions display higher levels of expression within wounded(POD14) sample.

Extended data Figure S14. Spatial in situ transcriptome analysis of Rnasel-/- versus wild type mice wounds

a.Integrated UMAP of *Rnasel-/-*(n=3) & wild type(n=3) mice wounds collected at scab detachment day 0 (SD0) with annotated clusters, a total of 33 clusters were automatically assembled, then manually annotated and grouped together. **b**. Heatmap of morphogenic, IL36 pathway immune and WNT genes within wound and non-wound areas *Rnasel-/-*(n=3) & wild type(n=3) (rows represent independent samples, color scale based on Z-score distribution) **c**. Graph-based clustering of *Rnasel-/-*(RLKO) wounds at SD0; (RLKO-3 same image displayed in Figure 7E) each colorcoded dot signifies a distinct cell type.(RLKO-1; bar = 2000 μm, RLKO-2; bar = 2000 μm, RLKO-3; bar = 1000 μm) **d**. Post-xenium histology image of *Rnasel-/-*(RLKO) wounds at SD0;

highlighted area represents determined wound area (RLKO-1; bar = 2000 μm, RLKO-2; bar = 2000 μm, RLKO-3; bar = 1000 μm). **e**. Graph-based clustering of wildtype (WT) wounds at SD0; (WT-2 same image displayed in Figure 7E) each color-coded dot signifies a distinct cell type.(WT-1; bar = 2000 μm, WT-2; bar = 1000 μm, WT-3; bar = 2000 μm) **f**. Post-xenium histology image of wildtype (WT)) wounds at SD0; highlighted area represents determined wound area (WT-1; bar = 2000 μm, WT-2; bar = 1000 μm, WT-3; bar = 2000 μm).

Supplementary Methods:

Colitis Model
In order to induce colitis, adult wild-type and *Il36r*$^{-/-}$ mice were fed up to 4% (w/v) 36-50kDa dextran sodium sulfate (DSS) (MP Biomedicals, 160110) in sterile water. Weight changes were monitored and recorded every day. For rescue experiments using the pan-caspase inhibitor Q-VD-OPh, mice were treated at the same concentrations as in WIHN experiments and were injected intraperitoneally during the day when noticeable weight loss began to occur (~day 3-4). Mice were eventually sacrificed and colon lengths were extracted for gross examination.

Retinoid derivatives measurements
All in vivo skin samples from wild-type and *Rnasel*$^{-/-}$ mice were collected and snap-frozen and stored at -80°C. Samples were then processed as described by *Kim et al* (1). Briefly, after homogenization, endogenous retinoids were extracted under low-intensity yellow light via dual-step liquid partitioning. Multistep partitioning was achieved using a highly selective liquid chromatography tandem mass spectrometry (LC-MS/MS) technique, LC-MRM(2). Retinoic acid measurements were performed using a Shimadzu Prominence ultra-fast liquid chromatograph ($UFLC_{XR}$) (Shimadzu, Columbia, MD) with AB Sciex 5500/6500+ hybrid triple quadrupole-linear ion trap (QqQ(LIT)) mass spectrometers (AB Sciex, Framingham, MA) using atmospheric pressure chemical ionization (APCI) conducted in pseudo-molecular MH+ mode. For all experiments, the reference controls used were 4,4-dimethyl-RA, retinyl acetate, and total retinyl ester for retinoic acid, retinol, and retinyl ester, respectively. Endogenous retinol and retinyl ester were measured and analyzed via UHPLC-UV using rapid resolution, reverse-phase Zorbax columns (SB-C18, Agilent) on a quaternary-based ACQUITY UPLC H-Class System (Waters Corporation, Milford, MA) with an ultraviolet detector.

Immunoblot analysis
Human and murine keratinocytes & mouse tissue were resuspended and lysed in M-PER Mammalian Protein Extraction Reagent (Thermo Fisher Scientific, 78501) containing an EDTA-based, broad-spectrum protease inhibitor cocktail (Thermo Fisher Scientific, 87786). Samples were then ultrasonically disrupted using a probe and then protein concentrations were quantified using the colorimetric BCA assay (Thermo Fisher Scientific, 23225). Proteins and ladder were loaded on to a denaturing NuPAGE Bis-Tris gel at a 4-12% gradient (Thermo Fisher Scientific, NP0321BOX) followed by electrophoresis. Proteins were then transferred and bound to a methanol-activated PVDF membrane (Bio-Rad, Hercules, CA). After a brief wash with 0.1% Tween-20 buffer, membranes were incubated in 5% non-fat dry milk (NFDM) blocking buffer for 1 hour at room temperature followed by an overnight incubation with primary antibody in blocking buffer at 4°C. Membranes were washed and incubated with corresponding horseradish peroxidase (HRP)-conjugated secondary antibodies at room temperature for 1 hour. All membranes were subsequently similarly probed for β-actin as a loading control for proteins. Proteins were detected on membranes using a luminol-based, HRP-reactive chemiluminescent substrate (Thermo Fisher Scientific, 34577) and visualized on a ChemiDoc XRS+ imaging system (Bio-Rad, Hercules, CA). Concentrated secreted protein from mouse keratinocytes were similarly processed. Mouse keratinocytes were disrupted in M- PER lysis buffer (Thermo Fisher Scientific, #78501) containing protease inhibitors (Thermo Fisher Scientific, #87786) using ultrasonic homogenizer (20% power with 5 times every 2 s) to extract proteins. Then, protein

concentrations were determined by BCA method (Thermo Fisher Scientific, #23225). Western blot procedures were followed by the protocol of NuPAGE system (Thermo Fisher Scientific). Briefly, 20 µg of protein samples were loaded for electrophoresis and transferred to polyvinylidene di-fluoride (PVDF) membrane (Bio-Rad, Hercules, CA). After blocking for at least an hour in 5% nonfat dry milk, the membrane was incubated with primary antibodies with appropriate dilutions (Supplementary table 2) at 4 °C overnight and followed by incubation with secondary antibodies for 1 h at room temperature. Protein amounts were normalized to rabbit polyclonal anti-human β- actin antibody (1:1000 dilution) (Cell Signaling Technology). Finally, proteins were visualized using SuperSignalTM West Pico PLUS chemiluminescent substrate kit (Thermo Fisher Scientific, #34577) and saved as image files using ChemiDoc XRS+ (Bio-Rad). The signal intensity of protein was quantified using Image LabTM software (Bio-Rad).

Immunofluorescence, immunocytochemistry, and immunohistochemistry
Immunofluorescence and fluorescence microscopy for mouse tissue was performed on de-paraffinized sections following heat-induced antigen retrieval using Target Retrieval Solution (Agilent Dako, S169984-2). After washing and permeabilization in TBS-T universal buffer (0.2% Triton X-100 in tris-buffered saline), sections were blocked at room-temperature in 5% goat, donkey or fetal bovine serum with 1% bovine serum albumin. Tissue sections were then incubated overnight at 4°C with primary antibodies at the suggested concentrations (Supplementary Table 3) in Antibody Diluent (Agilent Dako, S080983-2). Following subsequent washing, sections were incubated in fluorescent-dye conjugated secondary antibodies diluted in antibody diluent for 1 hour at room temperature. After final washing, sections were mounted with VECTASHIELD® Hardset™ Antifade Mounting Medium with DAPI (Vector Laboratories, H-1500) for nuclear staining. Human keratinocytes were prepared similarly, with the exception of antigen retrieval. All slides were imaged using either the DFC365FX (Leica) or Eclipse E-800 (Nikon) at 10x, 20x, and 40x magnifications.

siRNA transfection
For both mouse and human keratinocytes, cells were seeded at 50,000 cells/well (RNA) or 100,000 cells/well (protein) in 12-well or 6-well plates respectively. Non-targeting and gene-specific siRNAs (Dharmacon) (Supplementary Table 4) for human and mouse were used. Briefly, 25nM of siRNA was pooled with Lipofectamine® RNAiMAX transfection reagent (Thermo Fisher Scientific, 13778150) in reduced serum OPTI-MEM media (Gibco, 31985062) and added to cultured keratinocytes for 48 hours to achieve maximum gene knockdown efficiency.

1. Kim, D., *et al.* Noncoding dsRNA induces retinoic acid synthesis to stimulate hair follicle regeneration via TLR3. *Nature communications* **10**, 2811 (2019).
2. Brockes, J.P. Amphibian limb regeneration: rebuilding a complex structure. *Science* **276**, 81-87 (1997).
