# Supplementary material for: RNase L represses hair follicle regeneration through altered innate immune signaling
Source: J Clin Invest. 2025 Feb 4;135(6):e172595. doi: 10.1172/JCI172595 (PMC11910212; doi:10.1172/JCI172595)

Figure 4A

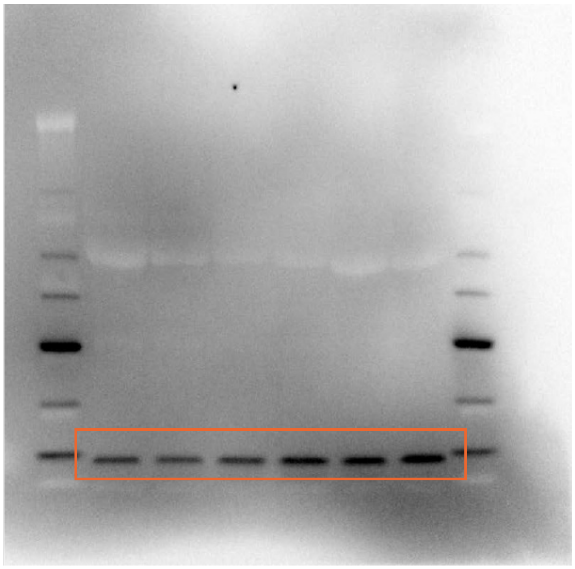

Il36α

β-actin

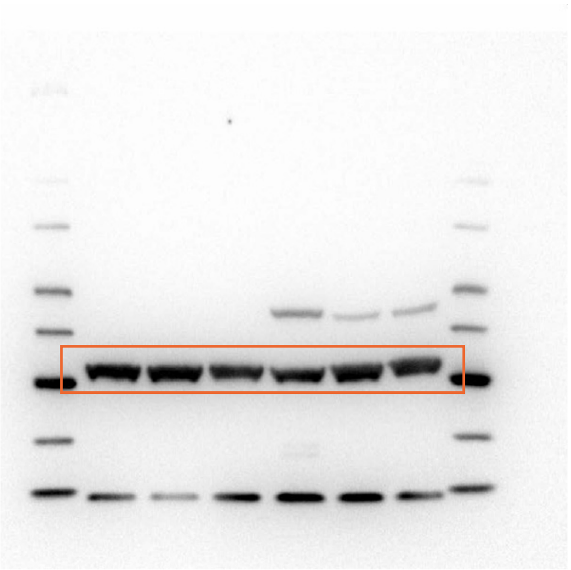

Figure 4B

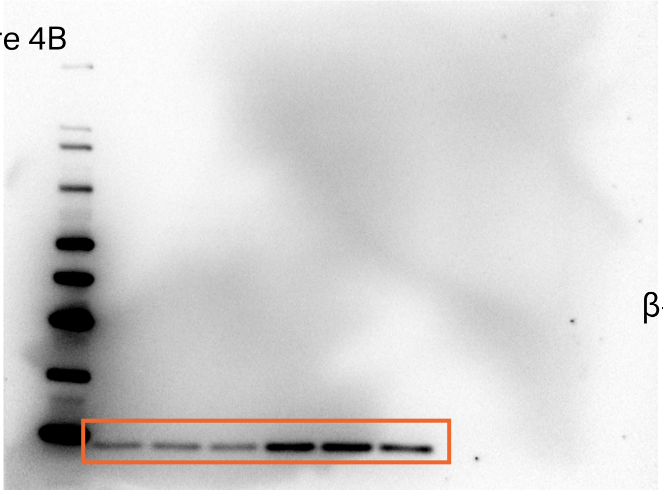

Il36α

β-actin

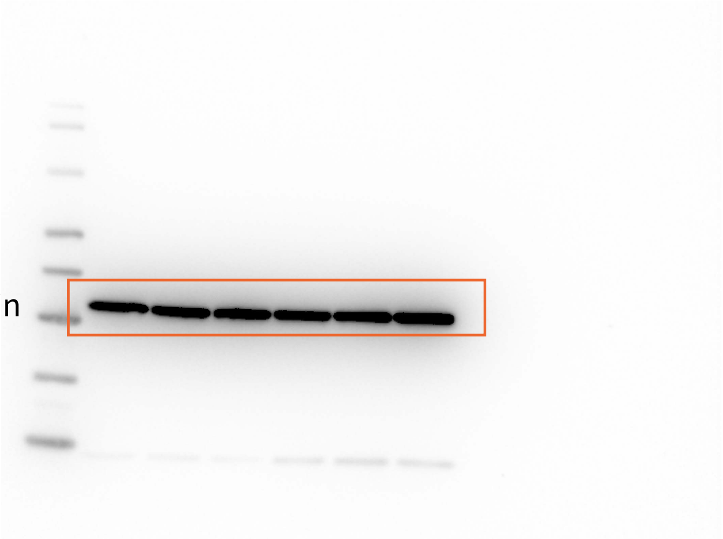

Figure 5D

β-actin

Il36rn

Il36α

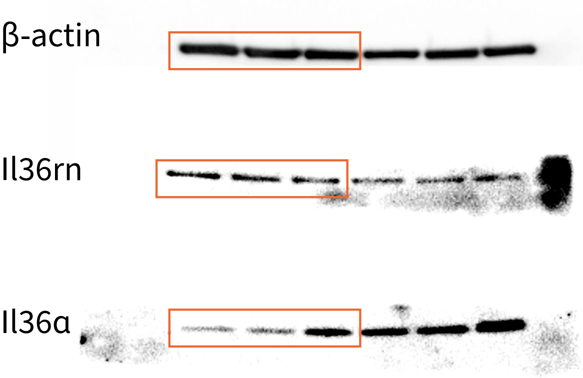

Figure 5D

β-actin

Il36rn

Il36α

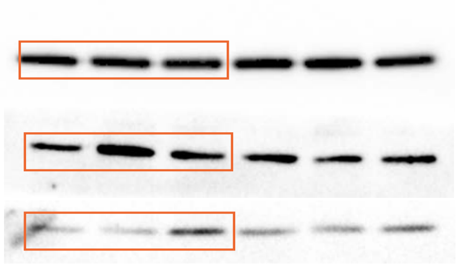

Figure 5D

β-actin

Il36rn

Il36α

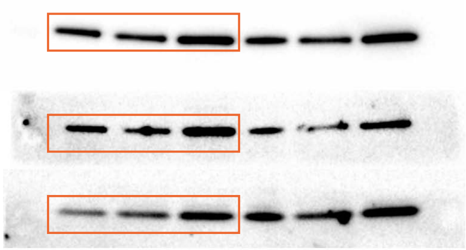

Figure 5E

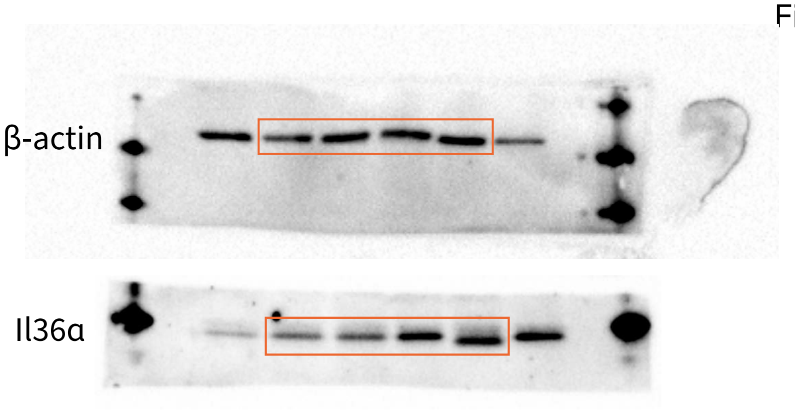

Figure 6C

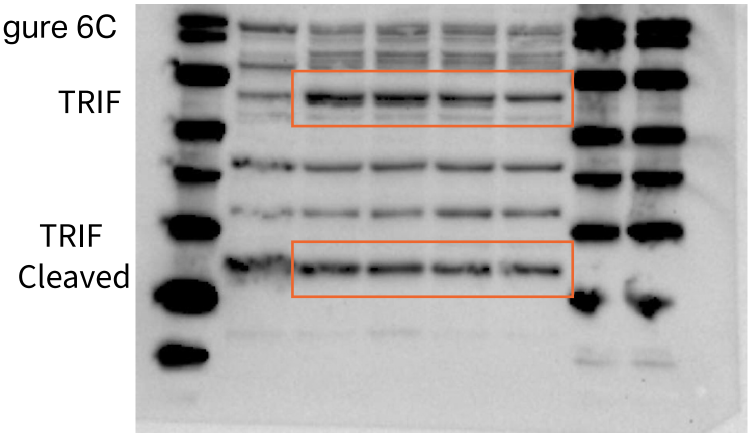

Figure 6D

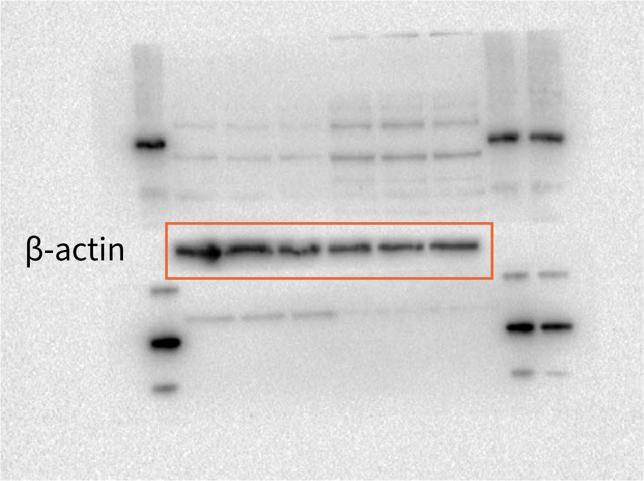

Figure 6C

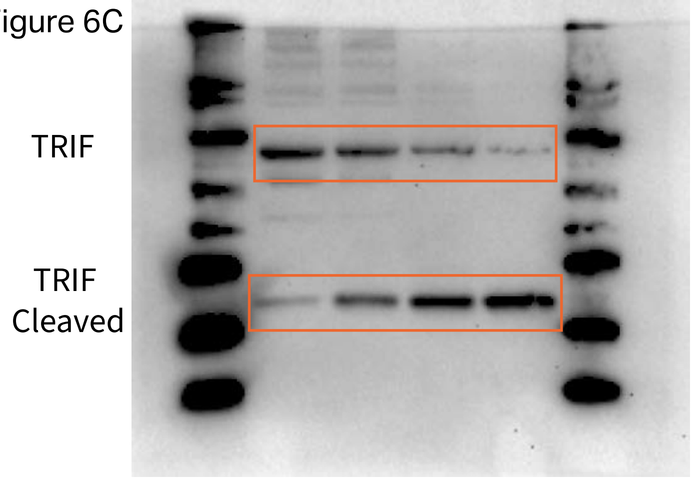

Figure 6D

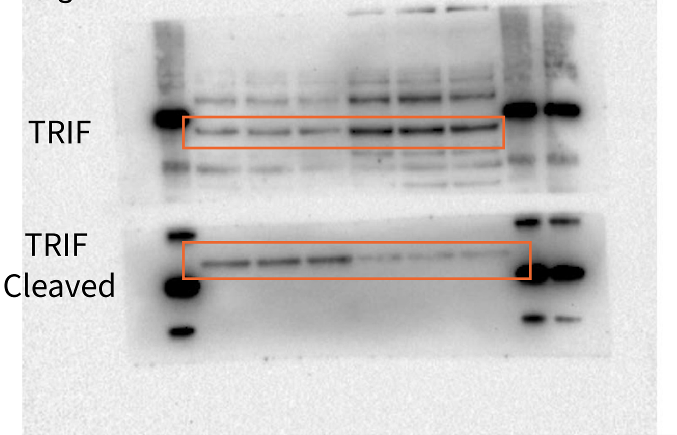

Figure 6F

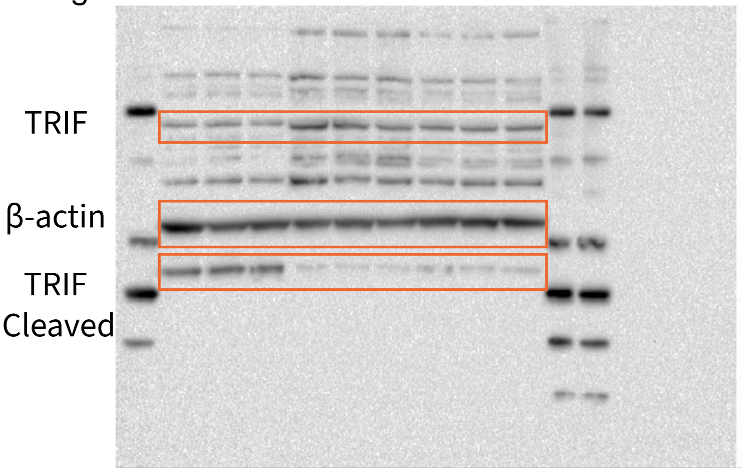

Figure 6H

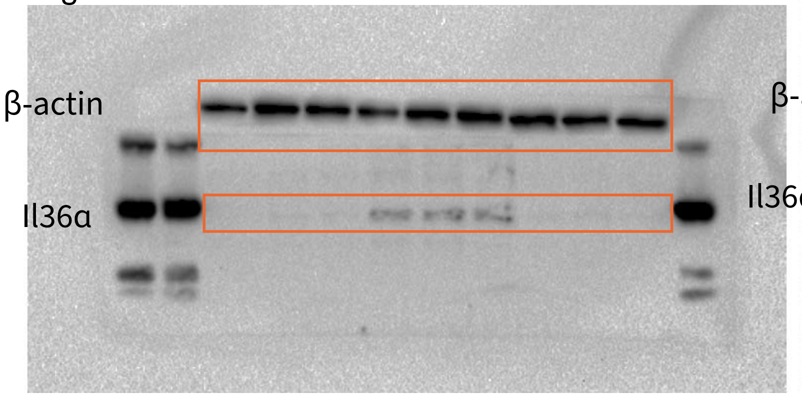

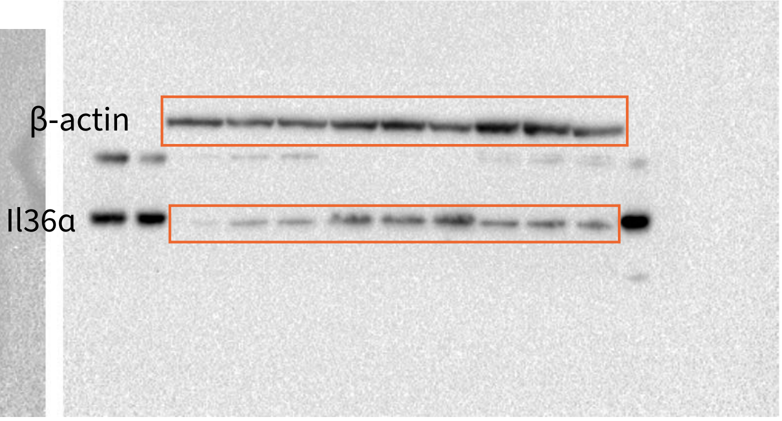

Supplemental S10A

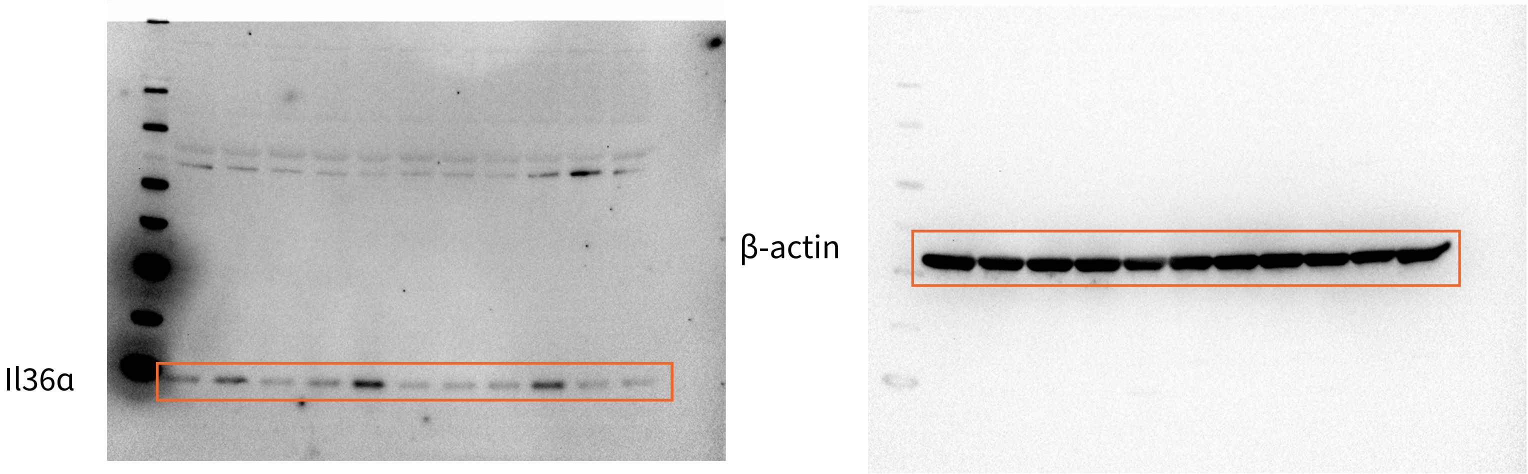

Supplemental S10C

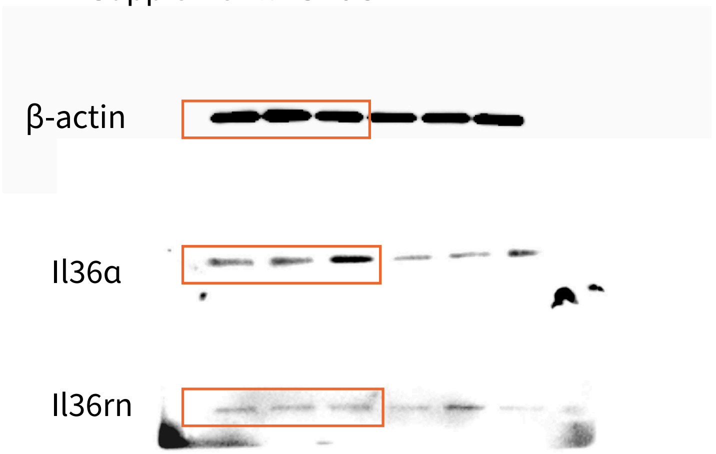

Supplemental S10D

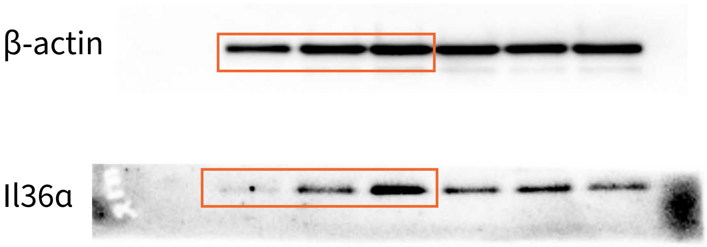

Supplement: Unedited blot and gel images [file jci-135-172595-s009.pdf]
